# A PET‐Surrogate Signature for the Interrogation of the Metabolic Status of Breast Cancers

**DOI:** 10.1002/advs.202308255

**Published:** 2024-05-17

**Authors:** Stefano Confalonieri, Bronislava Matoskova, Rosa Pennisi, Flavia Martino, Agnese De Mario, Giorgia Miloro, Francesca Montani, Luca Rotta, Mahila Esmeralda Ferrari, Laura Gilardi, Francesco Ceci, Chiara Maria Grana, Rosario Rizzuto, Cristina Mammucari, Pier Paolo Di Fiore, Letizia Lanzetti

**Affiliations:** ^1^ IEO European Institute of Oncology IRCCS Via Ripamonti 435 Milan 20141 Italy; ^2^ Department of Oncology University of Torino Medical School Candiolo Turin 10060 Italy; ^3^ Candiolo Cancer Institute FPO‐IRCCS Str. Provinciale 142 km 3.95, Candiolo Turin 10060 Italy; ^4^ Department of Biomedical Sciences University of Padua Via U. Bassi 58/B Padua 35131 Italy; ^5^ Department of Oncology and Haemato‐Oncology University of Milan Milan 20142 Italy

**Keywords:** breast cancer, FDG‐PET, gene signature, glycolysis, metabolism

## Abstract

Metabolic alterations in cancers can be exploited for diagnostic, prognostic, and therapeutic purposes. This is exemplified by 18F‐fluorodeoxyglucose (FDG)‐positron emission tomography (FDG‐PET), an imaging tool that relies on enhanced glucose uptake by tumors for diagnosis and staging. By performing transcriptomic analysis of breast cancer (BC) samples from patients stratified by FDG‐PET, a 54‐gene signature (PETsign) is identified that recapitulates FDG uptake. PETsign is independently prognostic of clinical outcome in luminal BCs, the most common and heterogeneous BC molecular subtype, which requires improved stratification criteria to guide therapeutic decision‐making. The prognostic power of PETsign is stable across independent BC cohorts and disease stages including the earliest BC stage, arguing that PETsign is an ab initio metabolic signature. Transcriptomic and metabolomic analysis of BC cells reveals that PETsign predicts enhanced glycolytic dependence and reduced reliance on fatty acid oxidation. Moreover, coamplification of PETsign genes occurs frequently in BC arguing for their causal role in pathogenesis. CXCL8 and EGFR signaling pathways feature strongly in PETsign, and their activation in BC cells causes a shift toward a glycolytic phenotype. Thus, PETsign serves as a molecular surrogate for FDG‐PET that could inform clinical management strategies for BC patients.

## Graphical abstract

A molecular signature, PETsign, is derived from breast cancer (BC) patients stratified by ‐ positron emission tomography.  PETsign recapitulates the metabolic activity of BCs and is an independent predictor of disease outcome. C‐X‐C motif chemokine ligand 8 (CXCL8) and epidermal growth factor receptor (EGFR) signaling pathways are prominent in PETsign, and their activation in BC cells causes a shift toward a glycolytic phenotype.

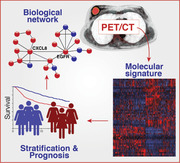

## 1 Introduction

The ability of tumors to adapt their metabolism to match specific needs is a recognized hallmark of cancer, crucial in shaping tumor evolution and disease progression.^[^ ^1^ ^]^ Metabolic plasticity is exemplified by aerobic glycolysis, commonly referred to as the Warburg effect.^[^ ^2^ ^]^ This phenomenon involves the avid uptake of glucose by cancer cells and its conversion to lactate even in the presence of oxygen.^[^ ^3^ ^]^ Traditionally, the Warburg effect was viewed as a strategy adopted by cancer cells to compensate for a deficit in energy production by mitochondria.^[^ ^4^ ^].^However, a more recent interpretation is that it serves to enhance glycolytic flux, thereby increasing the availability of metabolic intermediates to fuel anabolic pathways.^[^ ^3^, ^5^ ^]^

The Warburg effect can be indirectly studied in vivo using a variety of methods, the most common being 18F‐fluorodeoxyglucose (FDG)‐positron emission tomography (FDG‐PET). This imaging method is widely used in oncology for diagnosis, staging, re‐staging after therapy, and follow‐up. It relies on the use of the glucose‐derivative radiotracer, FDG, which is avidly taken by tumors characterized by increased glucose metabolism. PET imaging evaluates several parameters that provide different measurements of the extent of FDG uptake within a particular region (e.g., the tumor). Among them, the maximum standardized uptake value (SUVmax), corresponds to the highest FDG uptake value.^[^ ^6^ ^]^ Notably, SUVmax has been shown to correlate with tumor aggressiveness and, in some instances, to behave as an independent prognostic factor in different types of cancer, including breast, lung, and colon cancer.^[^ ^7^ ^]^ These findings underscore the importance of the Warburg effect in supporting tumor growth and progression.

Breast cancer (BC) is the most frequently diagnosed cancer worldwide, accounting for ≈12% of all cancer diagnoses and ≈7% of cancer‐related deaths annually.^[^ ^8^ ^]^ Several molecular subtypes of BC are routinely recognized and used in the clinic to predict prognosis and guide therapy‐decision making. These include: i) hormone receptor (HR, including estrogen receptor, ER, and progesterone receptor, PGR)‐positive (HR+) Luminal BCs, which can be further categorized into Luminal A or B based on the expression of the proliferation marker Ki67; ii) Luminal‐HER2 BCs which co‐express HR and the HER2 (also known as ERBB2) oncogene (HR+, HER2+); iii) HER2 BCs which are positive for HER2 but lack HR (HR‐, HER2+); iv) triple‐negative BCs (TNBCs) which lack expression of HR and HER2 (HR‐, HER2‐).^[^ ^9^ ^]^

This molecular subtyping serves as a prognostic indicator for disease aggressiveness and clinical outcome, with Luminal A having the most favorable prognosis, followed by Luminal B, Luminal‐HER2, HER2, and TNBC, in descending order. However, due to the significant heterogeneity in the molecular profiles and clinical behaviors of BCs,^[^ ^10^ ^]^ accurately classifying and treating them based on a limited number of molecular markers, in addition to standard clinicopathological parameters (e.g., tumor size, grade, node status, metastatic spread, age), does not always prove effective. As a result, much effort has been placed in the development of BC multigene signatures to predict prognosis and therapy response.^[^ ^11^ ^]^ These signatures are proving particularly valuable for Luminal BCs, which represent ≈65% of all BCs and exhibit considerable intertumoral heterogeneity. Although Luminal BCs are often associated with a favorable prognosis, approximately 20% of patients experience metastatic relapse within 10 years or later of surgery.^[^ ^12^ ^]^ Identifying these high‐risk patients is therefore crucial for selecting patients who are likely to benefit from more aggressive and prolonged therapies.

In BC, FDG‐PET imaging is a valuable tool for disease staging, typically employed from clinical stage IIB onward. For early‐stage BC its use is not routine due to its limited sensitivity for detecting lesions < 1.0 cm. In addition, FDG‐PET sensitivity can be limited by low tumoral FDG uptake. The main factors influencing uptake in BC are tumor grade, histological subtype, proliferation index, HR status and tumor phenotype. Grade 1/2 tumors, lobular carcinomas, low‐proliferative tumors and HR+ tumors show less FDG avidity, with higher rate of false negative results and subsequent reduced clinical utility of FDG‐PET/CT. This applies in particular to loco‐regional staging (T and N), for which an ultrasound and/or MRI evaluation is mandatory.^[^ ^13^ ^]^ However, metabolic profiling has the potential to provide valuable prognostic‐predictive information in all BC patients, irrespective of stage. Indeed, correlation between high SUVmax values and reduced BC patient survival has been reported, especially within the Luminal subtype.^[^ ^7^, ^14^ ^]^ Therefore, we hypothesized that a multigene signature recapitulating the SUVmax metabolic state could have prognostic value in BC patients. The present studies were undertaken to verify this hypothesis.

## 2 Results

### 2.1 Identification of a Molecular Signature Reflecting SUVmax Status in BCs

We analyzed a cohort of BC patients who had undergone FDG‐PET prior to any treatment, and were directed to surgery without receiving any neoadjuvant chemotherapy (mean time between FDG‐PET and surgery 21 ± 16 d, range 1–85 d). Patients were categorized based on their SUVmax, yielding two groups for our study: SUV‐High (SUV‐H, SUVmax > 10), and SUV‐Low (SUV‐L, SUVmax < 5) (Figure S1, Supporting Information). A total of 120 patients were selected for analysis (57 SUV‐H; 63 SUV‐L), constituting the “120‐PET cohort” (Table S1, Supporting Information).

**Figure 1:**
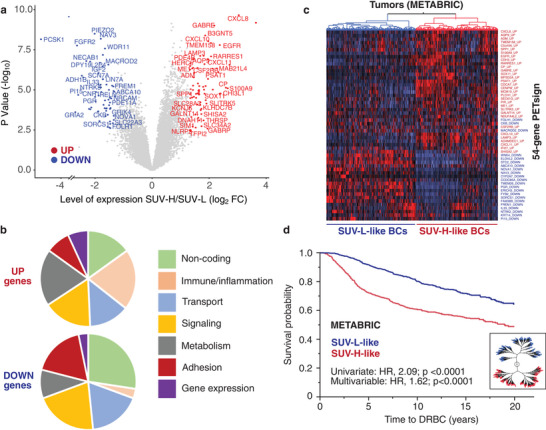
Derivation of the 54‐gene PETsign. a) Volcano plot showing the differentially expressed genes between SUV‐H and SUV‐L BCs. Significantly regulated genes (*p* < 0.05) are shown in red (upregulated) or blue (downregulated). b) Pie charts showing the functions associated with the 135 differentially expressed genes. Categories were attributed as described in Table S3 (Supporting Information). c) Hierarchical clustering of the METABRIC dataset by PETsign (54 genes). Columns, tumor samples; rows, genes (each labeled with its original UP or DOWN status, as from Table S7, Supporting Information). The dendrogram on the top shows the SUV‐H‐like (red) or SUV‐L‐like (blue) classification of the BCs in the dataset. d) The SUV‐H‐like and SUV‐L‐like BCs were subjected to KM analysis for time to DRBC (Death related to BC) in the METABRIC cohort. HR and *p*‐values (*p*) were calculated with the Cox proportional hazards model using JMP, in this and all other KM analyses shown. A constellation plot of tumor clustering (alternative representation of the data in c) is shown in the inset.

The transcriptomic profiles of the primary tumors from these patients were obtained by RNAseq of formalin‐fixed paraffin‐embedded (FFPE) samples, and their comparative analysis revealed 135 differentially expressed transcripts between SUV‐H versus SUV‐L tumors: 73 upregulated (FDR < 0.05, *p* < 0.05, FC > 2.5) and 62 downregulated (FDR < 0.05, *p* < 0.05, FC < 0.4) (**Figure** **1**a and Table S2, Supporting Information). Among these transcripts, 28 were noncoding RNAs (ncRNAs), while the remaining 107 were coding RNAs (Table S3, Supporting Information). Surprisingly, only a fraction of the coding genes could be clearly linked to “metabolic functions”, with many implicated in signaling, adhesion,membrane transport, and immune and inflammation pathways (Figure 1b). To ascertain whether the expression of these genes is linked to contamination of the tumor samples with immune and/or stromal cells, we performed a series of bioinformatics analyses. These controls revealed minimal contamination (Tables S4 and S5, Supporting Information), suggesting that the expression of immune/inflammation‐related genes likely originates from the epithelial component of the tumor.

### 2.2 Derivation of PETsign and Evaluation of Its Prognostic Value in BC Patients

To explore the potential clinical relevance of the identified genes, we interrogated the METABRIC database (Table S6, Supporting Information).^[^ ^15^ ^]^ Of the 135 differentially expressed transcripts between SUV‐H versus SUV‐L BCs, 99 were present in the METABRIC dataset, with most of the absent genes being ncRNAs. Fifty‐four of the 99 genes correlated with worse prognosis in univariate analysis (32 upregulated, 22 downregulated, Table S7, Supporting Information). These 54 prognostic genes constitute the “PET signature” (PETsign).

We used PETsign to stratify the METABRIC cohort and found that it clearly distinguished BCs resembling the SUV‐H‐like and SUV‐L‐like molecular phenotypes (Figure 1c and Table S8, Supporting Information). The SUV‐H‐like phenotype correlated with aggressive disease in both univariate [hazard ratio (HR), 2.09; *p* < 0.0001] and multivariable (HR, 1.62; *p* < 0.0001) analyses (Figure 1d and Table S8, Supporting Information). Thus, PETsign is a strong independent prognostic indicator of adverse disease outcomes. When applied to different molecular subtypes of BC, PETsign predicted poor prognosis in Luminal BCs (HR+, HER2‐), while no significant association was observed in other molecular subtypes (Table S8, Supporting Information).

### 2.3 Independent Validation of PETsign

The derivation of PETsign was based on selecting individually prognostic genes. Consequently, the stratification of the METABRIC cohort with PETsign might be influenced by overfitting. To address this concern, we evaluated the robustness of PETsign as a prognostic signature using two independent BC cohorts: The Cancer Genome Atlas (TCGA) cohort,^[^ ^16^ ^]^ and the 970‐IEO subcohort (Table S6, Supporting Information).

**Figure 2:**
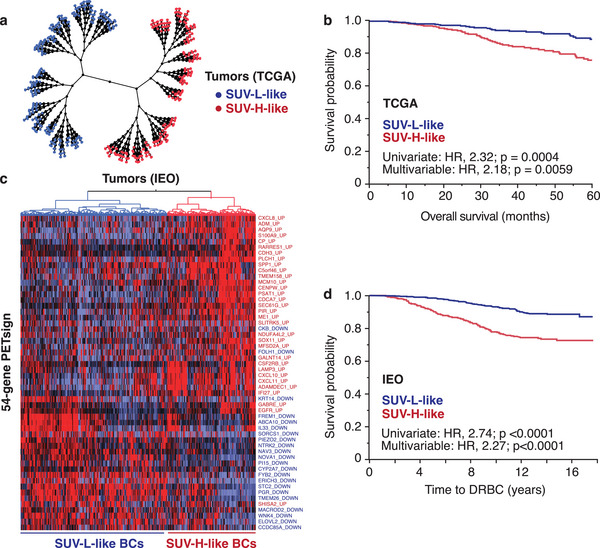
Validation of the prognostic value of PETsign in independent BC cohorts. a) Constellation plot showing the clustering of the TCGA cohort by PETsign. b) KM analysis of overall survival in the TCGA dataset. c) Hierarchical clustering of the 970‐IEO subcohort with the PETsign. Columns, tumor samples; rows, genes (each labeled with its original UP or DOWN status, as from Table S7, Supporting Information). The dendrogram on the top shows the SUV‐H‐like (red) or SUV‐L‐like (blue) classification of the BCs in the dataset. d) KM analysis in the 970‐IEO subcohort.

The TGCA dataset contains high quality molecular data, but limited clinical follow‐up: median follow‐up, 3.58 years (Table S6, Supporting Information). Therefore, we restricted our analysis of this cohort to a 5‐year follow‐up period. Within this timeframe, PETsign was an independent predictor of prognosis in univariate (HR, 2.32; *p* = 0.0004) and multivariable (HR, 2.18; *p* = 0.0059) analysis (**Figure** **2**a,b and Table S8, Supporting Information).

To achieve independent validation of PETsign in a high‐quality clinical cohort, we used a consecutive cohort of ≈2300 BC patients who underwent surgery at IEO from 1997 to 2000 (Table S9, Supporting Information).^[^ ^17^ ^]^ From this cohort, we selected 970 cases that matched the entire cohort for clinicopathological characteristics (Table S9, Supporting Information). Transcriptomic profiling by RNAseq was performed on the 970‐IEO subcohort. PETsign effectively distinguished tumors with a SUV‐H‐like and SUV‐L‐like molecular phenotype in this cohort (Figure 2c). The SUV‐H‐like phenotype correlated with poor prognosis in univariate (HR, 2.74, *p* < 0.0001) and multivariable (HR, 2.27, *p* < 0.0001) analysis (Figure 2d and Table S9, Supporting Information). Consistent with the METABRIC cohort, PETsign predicted poor prognosis specifically in Luminal BCs in the 970‐IEO subcohort (Table S9, Supporting Information). Although the prognostic value in Luminal A BCs could not be established due to the small number of events, PETsign correlated with poor prognosis in Luminal B BCs, the most common type of BC (Table S8, Supporting Information).

Thus, PETsign is a robust independent prognostic indicator of poor clinical outcome, validated across three independent BC cohorts that were generated using different technological platforms for transcriptomic profiling, in different institutes and countries.

### 2.4 PETsign Is an Ab Initio Signature of Aggressive Disease Course

**Figure 3:**
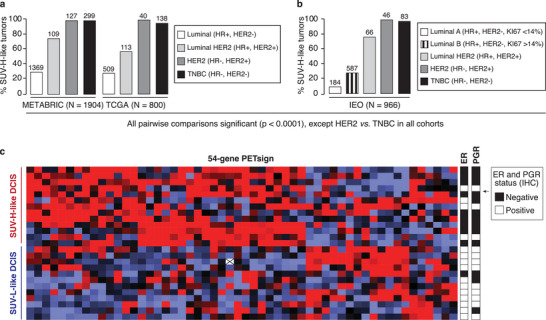
Association of the SUV‐H‐like phenotype with BC molecular subtypes and DCIS. a,b) The percentage of SUV‐H‐like tumors within each of the BC molecular subtypes is shown. The total number of tumors is indicated on the top of each bar. Due to the lack of complete clinicopathological information, 96 TCGA cohort cases and 4 IEO cohort cases were excluded from the analysis. The availability of Ki‐67 staining in the IEO cohort allowed the classification of Luminal A and B tumors. In a,b), *p*‐values were assessed by the chi‐square tests of significance using JMP. c) Twenty‐five DCIS (rows) were clustered using the PETsign genes (columns). The HR (ER/PGR) status of each DCIS, determined by IHC, is shown on the right.

To understand why PETsign was prognostic specifically in Luminal BCs, we examined the association of SUV‐H‐like phenotype with the different BC molecular subtypes in the three clinical cohorts. Aggressive HER2 and TNBC subtypes exhibited a SUV‐H‐like phenotype in nearly all cases (**Figure** **3**a,b). In contrast, within the various Luminal (HR+) subtypes, the proportion of SUV‐H‐like tumors correlated with disease aggressiveness. In particular, the most aggressive Luminal‐HER2 (HR+, HER2+) BCs exhibited a higher prevalence of the SUV‐H‐like molecular phenotype compared to the less aggressive Luminal subtype (HR+, HER2‐) (Figure 3a,b). Additionally, Luminal B BCs displayed a higher proportion of SUV‐H‐like tumors compared to the less aggressive Luminal A subtype (Figure 3b). These findings suggest that while the SUV‐H‐like phenotype is an inherent characteristic of aggressive BC subtypes (HER2, TNBC), within Luminal (HR+) BCs, it is associated with prognosis.

These data prompt the question of whether the SUV‐H‐like phenotype emerges from the outset (i.e., ab initio) or if it develops during BC progression. We analyzed a published cohort of DCIS,^[^ ^18^ ^]^ which represents the earliest stage of BC, characterized by noninvasive or preinvasive lesions confined within the ducts and not penetrating the basement membrane. In the DCIS cohort, PETsign effectively distinguished SUV‐H‐like and SUV‐L‐like subgroups (Figure 3c). In addition, most SUV‐H‐like DCIS lacked HR expression (Figure 3c). These findings indicate that the SUV‐H‐like molecular phenotype is present from the earliest stage of BC development, arguing that PETsign is an ab initio signature of the metabolic state of BCs.

### 2.5 PETsign Genes Are Frequently Coamplified in BC

**Figure 4:**
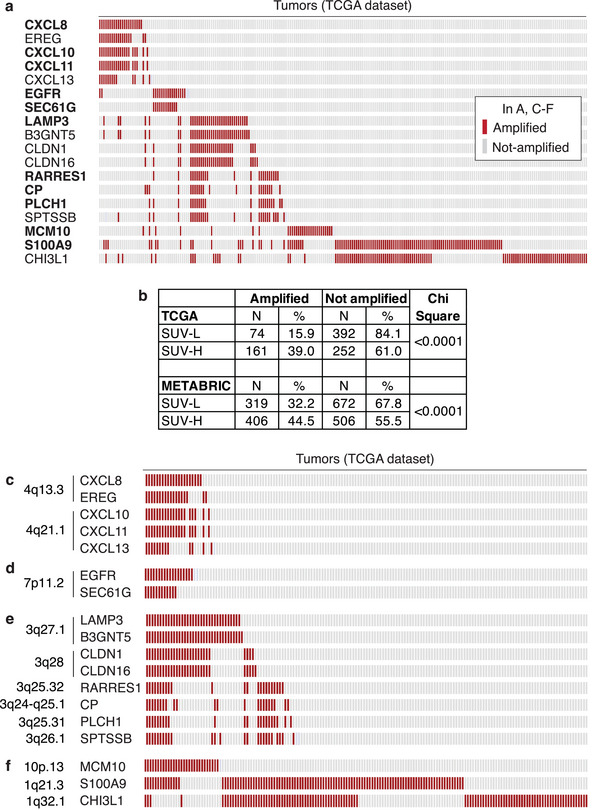
Coamplification of PETsign and 135‐signature genes in BC. a) Amplification of the 54 PETsign genes and the remaining upregulated genes from the original 135‐signature in the TCGA cohort. We identified 18 genes exhibiting amplification in at least 1.5% of cases and co‐occurrence with another amplified gene using a *q*‐value <0.001. In total, 235 TGCA BC cases (26%) harbored amplification/co‐amplification of the 18 genes (indicated by red bars). PETsign genes are in bold, while the other genes belong to the 135‐signature. b) Percentage of SUV‐H‐like and SUV‐L‐like tumors from the TCGA and METABRIC cohorts harboring amplification of one or more of the 18 genes. *P*‐values were calculated by the chi‐square test in Excel. *N* = number of patients in the various categories. c–f) Pattern of coamplification of the indicated groups of genes in the TCGA cohort. The chromosomal localization of each gene is indicated on the left.

In principle, the changes in gene expression identified by PETsign could either drive tumorigenesis or result from it. We examined the presence of genetic alterations in PETsign genes in SUV‐H‐like and SUV‐L‐like BCs. Analysis of the TCGA and the METABRIC databases uncovered rare mutations in PETsign genes in BC, with no significant recurrent mutations identified. However, examination of gene copy number in the TGCA database revealed an evident pattern of co‐amplification among 11 PETsign genes (**Figure** **4**a and Table S10, Supporting Information). We extended this analysis to the remaining upregulated genes from the 135 differentially expressed genes in SUV‐H versus SUV‐L tumors (see Tables S2 and S3, Supporting Information). This led to the identification of seven additional genes showing co‐amplification patterns (Figure 4a and Table S10, Supporting Information). The coamplification of these genes was confirmed in the METABRIC database (Figure S2a, Supporting Information). Overall, the 18 identified genes were cumulatively amplified in approximately one third of BCs (235/879 = 27%, in the TCGA, Figure 4a,b; 725/1903 = 38% in the METABRIC, Figure 4b and Figure S2a, Supporting Information). Interestingly, widening the analysis to include mRNA overexpression in addition to the amplification of the 18 genes, revealed that ≈56% of BCs (504/896 in the TCGA) showed one or both alterations (Figure S2b, Supporting Information).

We next asked whether a correlation exists between the SUV‐like molecular phenotype and the amplification of these 18 genes. We found that a higher percentage of SUV‐H‐like BCs exhibit amplification of one or more of the 18 genes compared with SUV‐L‐like BCs (Figure 4b). In addition, four groups of co‐amplified genes could be distinguished when the highest stringency criterion (q‐value for co‐occurrence < 0.001) was applied (Figure 4c–f and Table S10, Supporting Information). Three of the four groups are comprised of genes localized on the same chromosome: 4q, 7p or 3q, while the fourth group includes genes from two different chromosomes (Figure 4c–f).

Although the precise involvement of these gene amplifications/co‐amplifications in breast tumorigenesis remains to be established, the frequent amplification of PETsign genes in BCs argues for a causal role. This role could be associated with the heightened metabolic state observed in SUV‐H tumors and their increased clinical aggressiveness.

### 2.6 PETsign Stratifies BCs by Their Metabolic State

**Figure 5:**
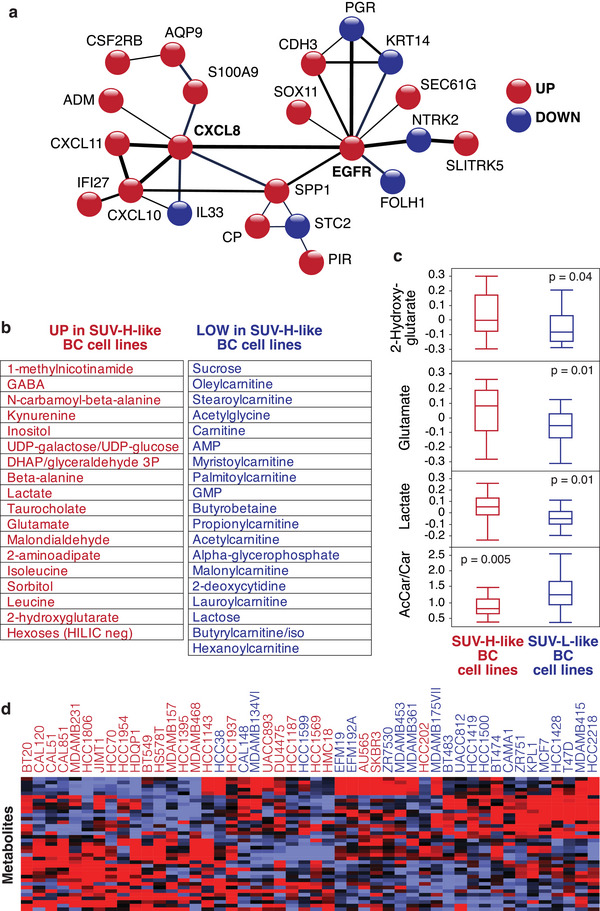
PETsign genes predict metabolic features of BC cell lines. a) STRING analysis (performed with default parameters) of PETsign proteins. Nonconnected nodes or poorly connected ones (1 edge) were removed. The thickness of the edges corresponds to the strength of the interaction. The color code indicates the direction of gene regulation in PETsign. b,c) Forty‐eight BC cell lines with available transcriptomic and metabolomic data were analyzed and metabolites with significantly different levels between SUV‐H‐like versus SUV‐L‐like cell lines were identified. b) List of significant differentially present metabolites (see also Table S11, Supporting Information). c) Boxplots of the levels of selected oncometabolites. AcCar/Car, acetylcarnitine to carnitine ratio. All *p*‐values were obtained by the non‐parametric Wilcoxon test using JMP. d) The metabolites (rows), identified in b, were used for unsupervised hierarchical clustering of the 48 BC cell lines (columns), yielding a grouping largely superimposable with the SUV‐H‐like and SUV‐L‐like phenotypes obtained by transcriptomics (see Figure S3, Supporting Information), indicated by red and blue cell line names, respectively.

To gain insights into the biological basis of PETsign, we performed a network analysis using the STRING database.^[^ ^19^ ^]^ Of the 54 PETsign proteins, 22 could be placed in a network centered around two major hubs: CXCL8 (C‐X‐C motif chemokine ligand 8) and EGFR (epidermal growth factor receptor) (**Figure** **5**a). The CXCL8 sub‐network is enriched in proteins involved in chemokine signaling pathways, while the EGFR sub‐network is enriched in proteins connected with signaling from the cell surface or hormonal response (see Table S3, Supporting Information, for protein function). This network analysis revealed an unexpected aspect of PETsign: its ability to stratify SUV‐H‐like and SUV‐L‐like BCs (and its prognostic value) appears to be driven by alterations in signaling pathways rather than expression of metabolic genes, in line with our initial analysis of the 135 differentially regulated genes (Figure 1b).

To investigate whether PETsign stratifies BCs based on their metabolic state, we utilized a panel of BC cell lines with publicly available transcriptomic and metabolomic datasets.^[^ ^20^ ^]^ PETsign was used to categorize the cell lines into SUV‐H‐like or SUV‐L‐like molecular phenotypes (Figure S3, Supporting Information). By comparing the metabolite levels in SUV‐H‐like versus SUV‐L‐like cell lines, we identified a group of metabolites significantly associated with the SUV‐H‐like molecular phenotype (Figure 5b and Table S11, Supporting Information). For instance, oncometabolites such as lactate, 2‐hydroxyglutarate, and glutamate were elevated in SUV‐H‐like versus SUV‐L‐like BC cell lines (Figure 5b,c). Notably, increased lactate production is a hallmark of the Warburg effect. Conversely, carnitine and its metabolites were decreased in SUV‐H‐like versus SUV‐L‐like BC cell lines (Figure 5b and Table S11, Supporting Information). In addition, the ratio of acetylcarnitine to carnitine was significantly lower in SUV‐H‐like cell lines, indicating that fatty acid oxidation is less active in these cell lines (Figure 5c). In essence, SUV‐H‐like cell lines appear to rely more on aerobic glycolysis, while SUV‐L‐like lines might depend more on fatty acid oxidation. Furthermore, unsupervised clustering demonstrated that the differentially produced metabolites can cluster BC cell lines according to their SUV‐like status (Figure 5d). Thus, PETsign directly correlates with the metabolic state of BC cell lines.

### 2.7 PETsign Genes Have a Causal Role in Shifting Metabolism toward Aerobic Glycolysis

We investigated whether PETsign genes exert a causal role in shifting their metabolism towards aerobic glycolysis. For this analysis, we required a cell model system in which we could determine whether activation of PETsign‐associated signaling—in particular CXCL8 and EGFR (see Figure 5a)—induces a shift towards a glycolytic phenotype. The “ideal” model would be a SUV‐L‐like BC cell line, exhibiting low glycolytic metabolism yet retaining the ability to respond to CXCL8 or EGFR signaling activation.

**Figure 6:**
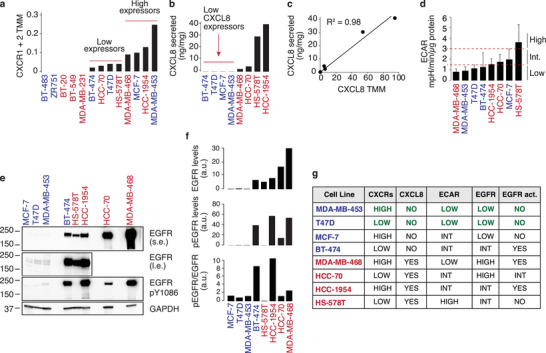
Characterization of CXCL8 and EGFR signaling pathways in a panel of BC cell lines. a) Expression of CXCR1 and CXCR2 by RNAseq in the indicated BC cell lines. CXCR1 and CXCR2 expression data are reported as combined (CXCR1 + CXCR2) Trimmed Mean of M‐values (TMM). SUV‐H‐like and SUV‐L‐like BC lines (see Figure S4a, Supporting Information) are shown in red and blue, respectively, in this and all other panels. b) CXCL8 secretion levels in the indicated BC cell lines. Results are the average of duplicate biological samples. c) Regression analysis of CXCL8 transcription (TMM, extracted from the RNAseq dataset) versus secretion (from panel b). d) ECAR (mpH/min/µg of protein), determined by Seahorse analysis. Data are expressed as the mean + SD of 3 independent experiments each with at least 4 technical replicates. Significance was calculated by the ANOVA one‐way test using SigmaPlot 14.0. Two arbitrary thresholds of 1.5 and 3 were used to stratify the cell lines as into low, intermediate and high ECAR groups. e) Immunoblot (IB) of the indicated cell lines with anti‐EGFR (s.e., short exposure; l.e., long exposure) and anti‐phosphoEGFR (EGFR‐pY1086). GAPDH, loading control. MW markers (kDa) are on the left. f) Densitometric quantitation of total EGFR and EGFR‐pY1086 (pEGFR) levels in the IB in (e). The ratio of pEGFR to EGFR is also shown. Data are expressed as arbitrary units (a.u.) after normalization to GAPDH values. g) Summary of data in panels a–f. The SUV‐like status of the cell lines is indicated (red, SUV‐H‐like; blue, SUV‐L‐like). MDA‐MB‐453 and T47D were chosen for subsequent experiments based on characteristics highlighted in green.

Given the known variability of cell lines across laboratories,^[^ ^21^ ^]^ we opted not to rely on publicly available datasets for identifying a SUV‐L‐like BC cell line. Instead, we performed transcriptomic profiling by RNAseq of a panel of 13 BC cell lines, representing all molecular subtypes of BC (Figure S4a, Supporting Information). Using PETsign, we categorized these cell lines according to their SUV‐like phenotype, identifying 6 SUV‐L‐like and 7 SUV‐H‐like BC cell lines (Figure S4a,b, Supporting Information). We assessed the competency of the cell lines for CXCL8 signaling. Eight cell lines expressed the CXCL8 receptors, CXCR1/CXCR2, to varying degrees (**Figure** **6**a). Subsequent testing for autocrine CXCL8 production in these eight cell lines revealed excellent concordance between CXCL8 secretion levels (Figure 6b) and mRNA expression levels (Figure 6c). Notably, four cell lines (BT‐474, T47D, MCF‐7, and MDA‐MB‐453) exhibited negligible secretion of CXCL8 despite expressing the cognate receptors. These cell lines, therefore, represent suitable models for conducting CXCL8 stimulation experiments, eliminating the confounding factor of endogenous CXCL8 secretion.

Subsequently, we analyzed the metabolic status of the 8 cell lines using Seahorse. Glycolytic activity was assessed by measuring the extracellular acidification rate (ECAR), an indicator of the rate of lactate production and the release of protons into the extracellular environment. Four cell lines (MDA‐MB‐468, MDA‐MB‐453, T47D and BT‐474) with a low ECAR and thus low glycolytic activity were identified (Figure 6d).

Finally, we determined the levels of EGFR expression and endogenous activation. Receptor activation was evaluated by measuring its phosphorylation levels with anti‐phosphoEGFR antibodies (Figure 6e and Figure S4c, Supporting Information). Three cell lines, MCF‐TR7, T47D, and MDA‐MB‐453, displayed low normal‐like levels of total EGFR and low levels of constitutive activation (Figure 6f), making them suitable models for EGF stimulation experiments.

Based on this characterization, we chose MDA‐MB‐453 and T47D to analyze the effects of CXCL8 and EGFR signaling activation on glycolysis. Both cell lines exhibit the SUV‐L‐like molecular phenotype, normal EGFR expression levels, low levels of constitutive EGFR activation, CXCR1/2 expression (although to different degrees), and negligible CXCL8 secretion (Figure 6g).

**Figure 7:**
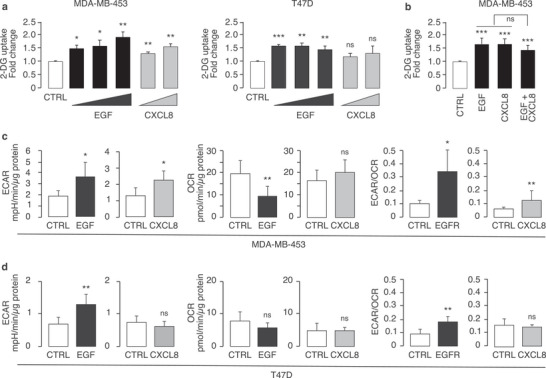
EGF and CXCL8 stimulation induces metabolic alterations in MDA‐MB‐453 and T47D. a) 2‐DG uptake in MDA‐MB‐453 (left) and T47D (right) cells treated with EGF (1, 10, or 100 ng mL^−1^ for 1 h) or CXCL8 (10 or 100 ng mL^−1^ for 1 h) or mock treated (CTRL). Data are expressed as mean fold‐increase over CTRL + SE (*n* = 4 independent biological replicas, with at least 4 technical replicates per experiment). Significance was calculated with the two‐tailed unpaired *t*‐test. b) 2‐DG uptake in MDA‐MB‐453 cells treated with EGF or CXCL8 alone or together (10 ng mL^−1^/each, 1 h). Data and significance are as in a (*n* = 3 independent biological replicas, each in sextuplicate). Note that all treatments are significant versus control, but EGFR+CXCL8 is not significant versus single treatments. c,d) Seahorse analysis of c) MDA‐MB‐453 cells and d) T47D cells, mock treated (CTRL) or treated with EGF or CXCL8 (100 ng mL^−1^ for 15 h). Results are the mean + SD of 3 independent experiments each with at least 4 technical replicates per experiment. The ECAR/OCR ratios are expressed as mpH/pmol and represent the mean + SD of ECAR/OCR values of 3 independent experiments with at least 4 technical replicates per experiment. Significance was calculated versus CTRL with the two‐tailed unpaired *t*‐test using SigmaPlot 14.0. In all panels: *p < 0.05, ** *p* < 0.01, *** *p* < 0.001, ns: not significant, versus CTRL.

We measured the uptake of 2‐deoxy‐glucose (2‐DG) following stimulation with EGF or CXCL8. EGF stimulation triggered a significant increase in 2‐DG uptake in both cell lines, while CXCL8 stimulation elicited this response only in MDA‐MB‐453 cells (**Figure** **7**a). The lack of response to CXCL8 in T47D cells is likely due to the low CXCR1/2 expression levels in these cells (Figure 6a). To investigate whether EGF and CXCL8 signaling might cooperate in inducing metabolic alterations, we measured 2‐DG uptake in MDA‐MB‐453 cells treated with the ligands alone and in combination. No additive effect of the combined treatment was observed (Figure 7b). This result suggests redundancy between the EGF and CXCL8 signaling, possibly due to their convergence on the same downstream pathways leading to the observed phenotype. The nature of these pathways remains to be elucidated, and might involve activation of PI3K/AKT signaling, as previously shown for regulation of glucose uptake by active EGFRs.^[^ ^22^ ^]^

To investigate whether the increased 2‐DG uptake was accompanied by a shift in metabolism towards aerobic glycolysis, we performed Seahorse analysis. Aerobic glycolysis is characterized by an increase in the glycolytic rate (measured by ECAR) relative to oxidative phosphorylation (measured by the oxygen consumption rate; OCR), resulting in an elevated ECAR to OCR ratio. Following EGF stimulation, we observed an increase in the ECAR/OCR ratio in both MDA‐MB‐453 and T47D cells (Figure 7c,d). Similarly, CXCL8 stimulation induced an increase in the ECAR/OCR ratio in MDA‐MB‐453 cells, while no effect was observed in T47D cells, reflecting the low CXCR1/2 expression levels in these cells (Figure 7c,d). These results suggest that EGF and CXCL8 stimulation can induce a shift towards aerobic glycolysis in SUV‐L‐like cells.

Overall, these findings support the idea that PETsign genes can play a causal role in determining the heightened glycolytic state observed in some BCs.

### 2.8 Combining PETsign with a Stem Cell Signature Enhances Its Prognostic Power

Our data indicates that PETsign captures the metabolic transcriptional landscape of BCs. We reasoned that combining PETsign with other signatures that recapitulate distinct aspects of BC biology could improve prognostication. As a proof of principle, we selected a 20‐gene signature developed in our lab, StemPrintER (SP).^[^ ^17a,c^ ^]^ SP interrogates the stemness traits of BCs which correlate with the size of the cancer stem cell compartment. Accordingly, BCs with a high SP status are characterized by adverse disease outcome.^[^ ^17a,c^ ^]^

PETsign and SP genes exhibit minimal overlap, with only 1 gene (CENPW) in common. While this observation can be attributed to the different biological traits (metabolism, stemness) captured by these signatures, an alternative explanation could be that the signature genes play a role in functionally overlapping but molecularly distinct pathways associated with the same biological trait.^[^ ^23^ ^]^ To investigate these possibilities, we investigated whether PETsign and SP are independently prognostic and therefore likely to explore different biological traits.

**Figure 8:**
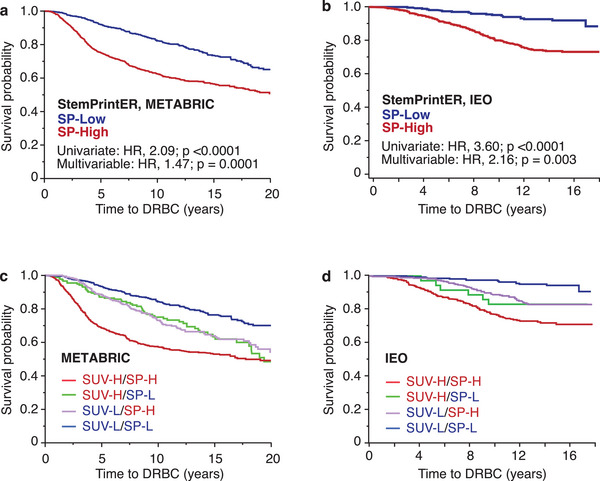
PETsign and StemPrintER are independent prognostic predictors. KM analysis in the a) METABRIC and b) 970‐IEO cohorts stratified by StemPrintER (SP). c,d) KM analysis in the c) METABRIC and d) 970‐IEO cohorts stratified by a combination of PETsign and SP genes. PETsign distinguishes SUV‐H‐like and SUV‐L‐like tumors, while SP identifies stem‐like SP‐High (SP‐H) and non‐stem‐like SP‐Low (SP‐L) tumors. See Table S12 (Supporting Information) for numerical details. HR and *p*‐values (*p*) were calculated by the Cox proportional hazards model using JMP.

Initially, we confirmed that SP, like PETsign, behaved as an independent predictor of disease outcome in the METABRIC and 970‐IEO cohorts (**Figure** **8**a,b). When PETsign and SP genes were used together, the prognostic power clearly increased in univariate analysis (Figure 8c,d and Table S12, Supporting Information). Kaplan‐Meier (KM) analyses revealed that a double‐low status (SUV‐L‐like/SP‐L) was associated with good prognosis, a single “high” status (either SUV‐H‐like or SP‐H) was associated with intermediate prognosis, and a double‐high status (SUV‐H‐like/SP‐H) was associated with the worst disease outcome (see Table S12, Supporting Information, for pairwise comparisons). In bivariate analysis, the PETsign and SP genes were independently prognostic of disease outcome in the complete METABRIC and 970‐IEO cohorts (including all molecular subtypes of BC), as well as in the subset of Luminal BCs in both cohorts (Table S12, Supporting Information).

Thus, PETsign and SP are independent prognostic signatures which, when combined, exhibit enhanced prognostic capability. This finding is consistent with the notion that they capture different biological traits: metabolism and stemness.

## 3 Discussion

We have developed and validated in independent BC cohorts a 54‐gene prognostic signature, PETsign, which stratifies tumors by their metabolic state: high or low SUVmax status. The SUV‐H‐like molecular phenotype almost invariably characterizes the most aggressive BC subtypes, TNBC and HER2. In the case of Luminal BCs, the most common and heterogeneous BC subtype, PETsign behaved as independent prognostic indicator, identifying those patients at greatest risk of adverse clinical outcome. This finding suggests that PETsign has the potential to guide clinical decision‐making by identifying Luminal BC patients most likely to benefit from aggressive and prolonged therapies.^[^ ^12^ ^]^

The identification of PETsign raises a number of biological and clinical questions. First, is PETsign a “true” metabolic signature, i.e., does it predict the metabolic status of BCs? Since PETsign was developed based on the categorization of tumors by FDG‐PET imaging, it suggests that it captures a transcriptional landscape associated with avid glucose uptake and increased glycolytic flux, indicative of aerobic glycolysis.^[^ ^24^ ^]^ Using a panel of BC cell lines, we demonstrated that the SUV‐H‐like and SUV‐L‐like molecular phenotypes distinguished by PETsign are associated with distinct metabolic profiles: SUV‐H‐like cell lines displayed increased lactate production (a hallmark of the Warburg effect) while SUV‐L‐like cell lines exhibited elevated carnitine metabolism indicative of increased reliance on fatty acid oxidation. In addition, activation of key signaling hubs identified in PETsign (EGFR and CXCL8) induced an increase in the rate of glycolysis relative to oxidative phosphorylation in BC cell lines. These observations support the notion that PETsign can identify aggressive BCs exhibiting metabolic reprogramming towards aerobic glycolysis.

Second, is the acquisition of the SUV‐H‐like molecular phenotype linked to the progressive adaptation of tumor cells to the changing metabolic demands imposed by microenvironmental conditions, such as presence of cytokines and growth factors, tumor:stroma interactions, hypoxic state, or nutrient availability,^[^ ^24^ ^]^ or does it represent an intrinsic feature of BCs present from the outset? Interestingly, examination of a DCIS cohort revealed that the SUV‐H‐like and SUV‐L‐like molecular phenotypes were already present at the earliest stage of BC. This finding suggests that metabolic rewiring towards aerobic glycolysis is an early alteration in tumorigenesis that directly contributes to an aggressive disease course.

Third, do PETsign genes have causal roles in determining aerobic glycolysis in BCs or are they regulated downstream of other alterations that are driving metabolic reprogramming? Our data support a causal role of at least some PETsign genes, as exemplified by CXCL8 and EGFR, whose activation induces metabolic modifications characteristic of aerobic glycolysis in a cell‐autonomous fashion. Moreover, clear patterns of coamplification of PETsign genes were observed primarily in SUV‐H‐like BCs, arguing for causality. It will be interesting to investigate potential cooperative roles of these amplified genes in metabolic reprograming. To this regard, we note that, while we report that some of the PETsign genes (CXCL8 and EGFR) might be involved in determining metabolic cell‐autonomous effects in BC, the role of non‐cell‐autonomous circuitries, involving tumor:stroma interactions and the establishment of hypoxic conditions, which play key roles in determining the glycolytic phenotype of tumors, was not addressed in our study, and remains therefore to be elucidated.

Finally, is there clinical utility for PETsign? We foresee three possible applications of PETsign. First, it could be used for prognostic stratification, something that will require benchmarking against other prognostic signatures. While we do not envision PETsign replacing existing multigene tests,^[^ ^11a,b,d^ ^]^ we believe that it could be successfully integrated with them to improve prognostication. In support of this notion, we demonstrate that PETsign can add significant prognostic information to StemPrintER.

Second, PETsign could be used to guide therapy decision‐making. A recent multiomics study in TNBC, identified three distinct “metabolic” subtypes: glycolytic, lipogenic, and mixed.^[^ ^25^ ^]^ These metabolic subtypes predicted both prognosis and sensitivity to therapies targeting the specific metabolic phenotype. Notably, the glycolytic subtype was associated with higher tumor grade, a basal‐like phenotype and worse clinical outcome.^[^ ^25^ ^]^ These findings together with our observation that the SUV‐H‐like phenotype correlates with enhanced glycolytic flux, suggest that PETsign could predict sensitivity to anti‐glycolytic drugs. Several such drugs are in preclinical and clinical development phases; PETsign could be instrumental in patient stratification for these clinical trials.^[^ ^26^ ^]^

Finally, PETsign could be used for the development of novel combinatorial therapy strategies for BC. TNBCs display frequent overexpression of the EGFR, in line with our observation that these tumors typically have a SUV‐H phenotype characterized by upregulation of this receptor.^[^ ^10^, ^27^ ^]^ However, targeted inhibition of the EGFR in TNBCs has largely failed in clinical trials.^[^ ^28^ ^]^ Our results suggest that EGFR has a redundant role, alongside CXCL8, in the metabolic reprogramming of BCs. If so, simultaneous targeting of these pathways might be necessary for therapeutic efficacy. Given that anti‐CXCL8 and anti‐CXCR1/2 drugs are in advanced stages of clinical development for the treatment of cancers and other diseases, combinatorial therapies involving these drugs and clinically available anti‐EGFR drugs are a real possibility.^[^ ^29^ ^]^

## 4 Experimental Section

### Experiments with BC Cell Lines

Culture conditions for the 13 BC cell lines are described in the legend to Figure S4 (Supporting Information). CXCL8 secretion was measured on the Luminex platform (Thermo Fisher) in biological duplicates on 24 h conditioned medium. Immunoblotting was performed on total cell lysates (30 µg) with: anti‐EGFR (EGFR806, in‐house, directed against the last 12 amino acids of EGFR), anti‐ERBB2 (Cell Signaling #2165), anti‐pEGFR‐pY1086 (Cell Signaling #2220), anti‐pEGFR‐pY1068 (Cell Signaling #3777), anti‐pEGFR‐pY992 (Cell Signaling #2235), anti‐GAPDH (Santa Cruz sc‐32233), anti‐actin (Merck A4700). Immunoblot data were collected using Chemidoc (Bio‐Rad) and the resulting‐.scn files were opened with the Image Lab software and converted into‐.tif images. For glucose uptake assays, cells were seeded in 96‐well plates (5000 cells per well). After 72 h of culture, the medium was replaced with complete medium containing, or not, EGF or CXCL8 for 1 h before measuring the uptake of 2‐DG with the Glucose Uptake‐Glo Assay kit (Promega), according to the manufacturer's instructions. OCR and ECAR analyses were performed with a seahorse XF24 extracellular flux analyzer (Agilent) as described.^[^ ^30^ ^]^ A titration with the uncoupler CCCP was performed to determine the CCCP concentration (1 × 10^−6^ m) that maximally increases OCR. The results were normalized for the protein content. In some experiments, 24 h after plating, cells were mock‐treated or treated with EGF (100 ng mL^−1^) or with CXCL8 (100 ng mL^−1^) in DMEM medium. After 15 h, medium was replaced with DMEM supplemented with 25 × 10^−3^ m glucose, 1 × 10^−3^ m sodium pyruvate, 30 × 10^−3^ m NaCl, 5 × 10^−3^ m HEPES, 1 × 10^−3^ m L‐glutamine, in the presence or absence of EGF or CXCL8, and measurements were performed.

### In‐House Clinical Cohorts and RNAseq

FFPE mammary tissue specimens were collected at IEO (Milan, Italy). All tissues were collected via standard operating procedures approved by the Institutional Ethical Board (reference: UID 2931), and informed consent was obtained for all tissue specimens linked with clinical data. The 120‐case PET cohort was collected at IEO (details are in Table S1, Supporting Information). For the present study, 120 BCs were selected which displayed a SUVmax > 10 (SUV‐H, *N* = 57) or a SUVmax < 5 (SUV‐L, *N* = 63). FFPE blocks were retrieved, and, after review of hematoxylin and eosin‐stained slides, areas of high tumor cellularity (>70%), devoid of DCIS, immune infiltrate and/or necrosis were identified. These areas were punched to extract a tissue core 1.5 mm in diameter and ≈2 mm in length, which was processed for RNA extraction. The IEO clinical cohort of ≈2000 consecutive BCs has been previously described (Table S6, Supporting Information);^[^ ^17c^ ^]^ the 970‐IEO subcohort used for RNAseq experiments is described in Table S9 (Supporting Information). For RNAseq, total cellular RNA was extracted and the quality was assessed using the Bioanalyzer 2100 (Agilent). Total RNA was depleted of ribosomal RNA and the RNAseq libraries were prepared with the Illumina TruSeq Stranded Total RNA kit. Following adapter ligation, libraries were amplified by PCR, checked on a Bioanalyzer 2100, quantified with picogreen reagent (Invitrogen), and sequenced for 100 bases in the paired‐end mode with 50 million reads coverage on a Novaseq 6000 sequencer. Raw data were acquired for all datasets, and the human reference genome (hg38) was employed as the alignment template for mapping the reads through Bowtie2 (version 2.4.5).^[^ ^31^ ^]^ The estimation of gene expression abundance was carried out using RSEM (version 1.3.3) with default parameters.^[^ ^32^ ^]^ RNAseq data that support the findings of this study are available on request from the corresponding author. The data are not publicly available due to privacy or ethical restrictions.

### Publicly Available Datasets

The TCGA BC dataset was downloaded from the cBioPortal (http://www.cbioportal.org/) (TCGA Breast Invasive Carcinoma. Source data from GDAC Firehose; previously known as TCGA Provisional).^[^ ^33^ ^]^ Only 896 M0 patients (896 cases) were analyzed, available as RSEM upper quartile normalized counts. The METABRIC dataset (1904 samples) was obtained through the cBioPortal (2019 freeze, available at https://github.com/cBioPortal/datahub/tree/master/public/brca_metabric).^[^ ^15^ ^]^ Data were available as normalized log2 intensity values.

Raw RNASeq data for the DCIS dataset were downloaded from the GEO database,^[^ ^18^ ^]^ accession number GSE69994. RNASeq and metabolomics data for BC cell lines were obtained from the Cancer Cell Line Encyclopedia (CCLE) collection (https://sites.broadinstitute.org/ccle/datasets). Cell line metabolomics data were available as log10 transformed data. Methodologies are to be found in the original publications.^[^ ^20^ ^]^ When raw RNASeq data were available, they were processed with RSEM (version 1.3.3) using Bowtie2 (version 2.4.5) as aligner and the human genome (hg38) as reference.

### Differential Expression Analysis between SUV‐H and SUV‐L Tumors

Following RNAseq, RNA counts were measured using the RESM software (version 1.3.3), and the unprocessed data were brought into the EdgeR package within the R software (version 3.40.2).^[^ ^34^ ^]^ Using default parameters, after filtering for not expressed or low expressed genes, library sizes were normalized and statistical analyses between groups were performed with the quasi‐likelihood F‐tests (QLF). Differentially expressed genes were obtained and the *p*‐value adjusted with the Benjamini and Hochberg methodology to obtain the FDR (false discovery rate). Only genes with an FDR *p*‐value < 0.05 and a fold‐change ≥ 2.5 were considered significant. Three genes, encoding ribosomal RNAs were excluded, as they likely represented contaminants, to yield the 135 initial gene list.

### Data Normalization, Hierarchical Clustering, and Survival Analysis

For hierarchical clustering and generation of heatmaps, when raw read counts were available, data (RNAseq data) were processed for TMM normalization with the EdgeR R package (version 3.40.2) and the Z‐Score was normalized using JMP software version 14.3 (version used for all analyses performed in JMP; SAS Institute Inc., Cary, NC, 1989–2023). Data that had previously been normalized (specifically, cBioPortal TCGA data) were log transformed and mean‐centered normalized. Metabolomics data, represented as log10 intensity values, or METABRIC gene expression data, represented as normalized log2 intensity, were solely subjected to mean‐centering. All Distance‐based dendrograms were created using the Ward's method in cluster analysis within JMP. Each sample cluster was categorized as either SUV‐H‐like or SUV‐L‐like based on the gene expression pattern of the 54 genes of PETsign genes. KM analyses, univariate and multivariable survival analyses were performed within JMP, employing the Survival platform and the Cox proportional hazards model, as appropriate. For the analyses involving the Cox proportional hazards regression model of the entire METABRIC dataset, shown in Table S7 (Supporting Information), the expression of each gene was categorized as HIGH or LOW with respect to the mean expression across all samples. Following this categorization, data were analyzed with the “survival” package in R, version 3.5‐5. The “coxph” function was employed to determine HR and *p*‐values for the univariate analysis (see also the legend of Table S7, Supporting Information, for further details).

### Other Statistical Analyses

For Seahorse measurements, statistics were calculated by using SigmaPlot 14.0. All results are representative of at least 3 independent experiments and are presented as the mean + SD. Significance was calculated by the ANOVA or the two‐tailed unpaired *t*‐test. *P*‐values < 0.05 were considered statistically significant. For the glucose uptake experiments, statistical analyses were performed using the two‐tailed unpaired *t*‐test. The results are expressed as mean + standard error (SE).

## Conflict of Interest

A patent application has been submitted for PETsign.

## Author Contributions

S.C., B.M., and R.P. contributed equally to this work. P.P.D.F. and L.L. are co‐last authors. Conceptualization: L.L., P.P.D.F. Investigation and data analysis: S.C., B.M., R.P., F.M., A.D.M., G.M., F.M., L.R., C.M. Clinical work: M.E.F., L.G., F.C., C.M.G. Funding acquisition: L.L., P.P.D.F., R.R. Supervision: L.L., P.P.D.F., R.R., C.M., F.C., C.M.G. Writing—original draft: L.L., P.P.D.F. Writing—review & editing: All authors.

## Supporting information

Supporting Information

## Data Availability

The data that support the findings of this study are available on request from the corresponding author. The data are not publicly available due to privacy or ethical restrictions.
