# Supplementary material for: A PET‐Surrogate Signature for the Interrogation of the Metabolic Status of Breast Cancers
Source: Adv Sci (Weinh). 2024 May 17;11(28):2308255. doi: 10.1002/advs.202308255 (PMC11267279; doi:10.1002/advs.202308255)
Supplement: Supplementary file 1 — Supporting Information [file ADVS-11-2308255-s001.docx]

Supporting Information

**A PET-surrogate Signature for the Interrogation of the Metabolic Status of Breast Cancers**

*Stefano Confalonieri*^#^*, Bronislava Matoskova*^#^*, Rosa Pennisi*^#^*, Flavia Martino, Agnese De Mario, Giorgia Miloro, Francesca Montani, Luca Rotta, Mahila Esmeralda Ferrari, Laura Gilardi, Francesco Ceci, Chiara Maria Grana, Rosario Rizzuto, Cristina Mammucari, Pier Paolo Di Fiore* and Letizia Lanzetti**

^#^ Contributed equally

*** Equal last authors

E-mail: Letizia Lanzetti (letizia.lanzetti@ircc.it), Pier Paolo Di Fiore (pierpaolo.difiore@ieo.it).

**SUPPORTING INFORMATION: TABLES**

**Table S1. Clinicopathological characteristics of the 120-PET cohort.**

|  | **N** | **SUV-Low (N)** | **SUV-High (N)** | **SUV-Low (%)** | **SUV-High (%)** | **χ2 p-Value** |
| --- | --- | --- | --- | --- | --- | --- |
| Samples | 120 | 63 | 57 | 52.50% | 47.50% |  |
| Age <50 | 69 | 32 | 37 | 46.38% | 53.62% | 0.12 |
| Age ≥50 | 51 | 31 | 20 | 60.78% | 39.22% |  |
| pT1-2 | 91 | 48 | 43 | 52.75% | 46.15% | 0.92 |
| pT3 | 29 | 15 | 14 | 51.72% | 48.28% |  |
| M0 | 112 | 57 | 55 | 50.89% | 49.11% | 0.19 |
| M1 | 8 | 6 | 2 | 75.00% | 25.00% |  |
| pN Neg | 21 | 6 | 15 | 28.57% | 71.43% | 0.13 |
| pN Pos | 98 | 57 | 41 | 58.16% | 41.84% |  |
| HR Pos | 98 | 56 | 42 | 57.14% | 42.86% | 0.03 |
| HR Neg | 22 | 7 | 15 | 31.82% | 68.18% |  |
| HER2 AMP | 22 | 10 | 12 | 45.45% | 54.55% | 0.47 |
| HER2 WT | 98 | 53 | 45 | 54.08% | 45.92% |  |
| KI67 <15% | 9 | 7 | 2 | 77.78% | 22.22% | 0.11 |
| KI67 ≥15% | 111 | 56 | 55 | 50.45% | 49.55% |  |

**Table S1.** Comparison of the clinicopathological characteristics of the SUV-High (SUVmax > 10) and SUV-Low (SUVmax < 5) BC patients of the 120-PET cohort. P-values were assessed by the chi-square tests of significance with JMP.

Methods: The 120-case PET cohort was collected at IEO (Milan, Italy) and selected out of a larger cohort of 244 patients. Medical records were screened for all patients with newly diagnosed BC who underwent 18F-FDG PET/CT before any treatment between December 2010 and March 2016. cT1-T3 BC patients were enrolled, excluding patients with multicentric disease. PET/CT scans were carried out with 3D PET/CT scanner and iterative reconstruction (GE Healthcare, Milwaukee, Winsconsin, USA) after the intravenous administration of 3.5 MBq/Kg of 18F-FDG. PET data were acquired from the skull base to the mid-thigh in three-dimensional mode and a scan time of 2.5-3 minutes per bed position. The tomographic raw data were corrected for attenuation using transmission data derived from low-dose non-contrast CT scans performed before emission imaging according to a standardized protocol with the following settings: 120 KVp, 80 mA, tube rotation 0.8 sec, table speed 15 mm/sec, pitch 1.5. For visual interpretation, images were displayed in the three orthogonal projections and as whole-body maximum-pixel-intensity projection (MIP) images. Visual analysis was performed on digital transaxial PET, CT, and fused images. Radiotracer uptake was considered abnormal if it was focal and appreciable in two or more slices, unexplainable by physiological or benign/inflammatory processes, and of higher intensity compared to surrounding tissues. Suspicion of malignancy was based on the combined analysis of PET, co-registered CT, and fused PET/CT images. For quantitative analysis, the slice with highest uptake was selected on axial images. For the present study, the region of interest (ROI) was defined manually around the lesion by one operator (MEF) and validated by another operator (LG), to avoid heterogeneity of ROI profiling due to distinct operators, and the single-pixel maximum standardized uptake value (SUVmax) was determined.

The SUVmax cut-offs (<5 and >10) were selected based on the following considerations: in a survey conducted on 20 primary studies with 3115 BC patients, SUVmax values above 5.55 were found to be a significant risk factor for both the event free survival and the overall survival in BC patients.^[1]^ Thus, a threshold of 5 was hypothesized (for the purpose of patients’ selection for the study) to represent a suitable cut-off for a low-risk situation. Accordingly, we selected a value twice as large (10) as the higher cut-off, to maximize the possibility of selecting two groups distant enough for the subsequent molecular analyses, as also supported by studies showing that SUVmax values around 10 correlate with increased risk of disease relapse.^[2]^ As a result, from the original cohort of 244 BC patients, we selected 120 patients, 63 displaying SUVmax <5 (SUV-Low) and 57 displaying SUVmax >10 (SUV-High).

**Table S2. List of the 135 genes differentially expressed between SUV-H *vs.* SUV-L BCs of the 120-PET cohort.**

| **Gene Name** | **SUV-H**  ***vs*.**  **SUV-L** | **FC** | **p-value** | **FDR** |
| --- | --- | --- | --- | --- |
| LOC105371822 | UP | 12.47 | <0.001 | 0.001 |
| CXCL8 | UP | 8.22 | <0.001 | 0.001 |
| TGFB2-OT1 | UP | 6.57 | <0.001 | <0.001 |
| S100A9 | UP | 6.28 | <0.001 | 0.005 |
| CP | UP | 6.01 | <0.001 | <0.001 |
| CHI3L1 | UP | 5.29 | <0.001 | <0.001 |
| EGFR | UP | 5.18 | <0.001 | <0.001 |
| LOC105374557 | UP | 5.17 | <0.001 | <0.001 |
| MAB21L4 | UP | 4.99 | <0.001 | 0.001 |
| ATP8A2 | UP | 4.83 | <0.001 | 0.001 |
| GABRE | UP | 4.54 | <0.001 | 0.002 |
| MMP1 | UP | 4.38 | <0.001 | 0.001 |
| LOC112268106 | UP | 4.36 | <0.001 | 0.001 |
| RARRES1 | UP | 4.19 | <0.001 | 0.001 |
| TMEM158 | UP | 4.18 | <0.001 | <0.001 |
| CXCL10 | UP | 3.91 | <0.001 | 0.001 |
| PSAT1 | UP | 3.89 | <0.001 | 0.009 |
| CXCL11 | UP | 3.89 | <0.001 | 0.001 |
| GABRP | UP | 3.84 | <0.001 | 0.002 |
| SLITRK5 | UP | 3.75 | <0.001 | 0.006 |
| SOX11 | UP | 3.68 | <0.001 | 0.001 |
| EREG | UP | 3.67 | <0.001 | <0.001 |
| B3GNT5 | UP | 3.64 | <0.001 | <0.001 |
| CXCL13 | UP | 3.58 | <0.001 | <0.001 |
| AQP9 | UP | 3.57 | <0.001 | 0.001 |
| HPGD | UP | 3.53 | <0.001 | <0.001 |
| PAK3 | UP | 3.50 | <0.001 | 0.001 |
| THRSP | UP | 3.49 | <0.001 | 0.005 |
| NDUFA4L2 | UP | 3.46 | 0.001 | 0.016 |
| SLC34A2 | UP | 3.32 | <0.001 | <0.001 |
| UBD | UP | 3.31 | <0.001 | <0.001 |
| KLHDC7B | UP | 3.31 | <0.001 | 0.004 |
| SPTSSB | UP | 3.26 | <0.001 | 0.001 |
| CLDN16 | UP | 3.24 | <0.001 | 0.001 |
| LAMP3 | UP | 3.22 | <0.001 | 0.005 |
| SLC28A3 | UP | 3.22 | <0.001 | <0.001 |
| PLCH1 | UP | 3.15 | 0.001 | 0.022 |
| DNAH11 | UP | 3.14 | <0.001 | 0.001 |
| EPOP | UP | 3.14 | <0.001 | 0.002 |
| ADAMDEC1 | UP | 3.13 | <0.001 | <0.001 |
| CLDN1 | UP | 3.12 | 0.003 | 0.034 |
| MFSD2A | UP | 3.11 | <0.001 | 0.011 |
| CDCA7 | UP | 3.11 | <0.001 | 0.007 |
| C5orf46 | UP | 3.09 | <0.001 | 0.010 |
| SHISA2 | UP | 3.07 | <0.001 | <0.001 |
| LOC105375172 | UP | 3.05 | <0.001 | 0.001 |
| LINC00673 | UP | 3.02 | <0.001 | 0.002 |
| LOC107987296 | UP | 3.02 | <0.001 | <0.001 |
| CENPW | UP | 3.02 | 0.002 | 0.031 |
| LOC105375170 | UP | 2.92 | <0.001 | 0.001 |
| CCDC144NL-AS1 | UP | 2.91 | <0.001 | 0.001 |
| IGHG4 | UP | 2.88 | <0.001 | 0.005 |
| LOC107986350 | UP | 2.86 | <0.001 | 0.009 |
| CSF2RB | UP | 2.77 | <0.001 | 0.007 |
| IFI27 | UP | 2.76 | 0.002 | 0.029 |
| ADM | UP | 2.76 | <0.001 | <0.001 |
| PDE4B | UP | 2.71 | <0.001 | 0.001 |
| SEC61G | UP | 2.71 | <0.001 | 0.001 |
| CDH3 | UP | 2.71 | <0.001 | 0.001 |
| LOC105376442 | UP | 2.70 | <0.001 | 0.001 |
| SIM1 | UP | 2.68 | <0.001 | 0.009 |
| PIR | UP | 2.65 | <0.001 | <0.001 |
| MOXD1 | UP | 2.65 | <0.001 | 0.005 |
| GALNT14 | UP | 2.59 | <0.001 | <0.001 |
| TFPI2 | UP | 2.55 | <0.001 | 0.001 |
| CYP1B1 | UP | 2.55 | 0.002 | 0.031 |
| HERC5 | UP | 2.55 | <0.001 | 0.001 |
| SPP1 | UP | 2.53 | <0.001 | 0.002 |
| NLRP2 | UP | 2.52 | <0.001 | 0.001 |
| MCM10 | UP | 2.52 | 0.002 | 0.032 |
| ME1 | UP | 2.52 | <0.001 | <0.001 |
| PRR16 | UP | 2.51 | <0.001 | <0.001 |
| KCNJ6 | UP | 2.50 | <0.001 | 0.005 |
| ABCA10 | DOWN | 0.40 | 0.001 | 0.020 |
| SLC22A3 | DOWN | 0.39 | <0.001 | 0.001 |
| GRIK4 | DOWN | 0.39 | <0.001 | <0.001 |
| ME3-DT | DOWN | 0.39 | <0.001 | 0.002 |
| FREM1 | DOWN | 0.39 | <0.001 | 0.003 |
| LOC105375896 | DOWN | 0.39 | <0.001 | 0.009 |
| LINC01016 | DOWN | 0.38 | <0.001 | 0.003 |
| CCDC158 | DOWN | 0.37 | <0.001 | <0.001 |
| CYP2A7 | DOWN | 0.36 | <0.001 | <0.001 |
| LOC105375387 | DOWN | 0.36 | <0.001 | <0.001 |
| FOLH1 | DOWN | 0.36 | <0.001 | <0.001 |
| ADIPOQ | DOWN | 0.36 | <0.001 | <0.001 |
| NOVA1 | DOWN | 0.36 | <0.001 | <0.001 |
| PDE11A | DOWN | 0.36 | <0.001 | 0.001 |
| NRCAM | DOWN | 0.36 | <0.001 | 0.001 |
| LOC105374020 | DOWN | 0.36 | <0.001 | 0.001 |
| TMEM26 | DOWN | 0.35 | <0.001 | 0.002 |
| CCDC85A | DOWN | 0.35 | <0.001 | <0.001 |
| RELN | DOWN | 0.35 | <0.001 | 0.001 |
| LOC105378747 | DOWN | 0.35 | <0.001 | <0.001 |
| LOC101929322 | DOWN | 0.34 | 0.003 | 0.040 |
| KRT15 | DOWN | 0.34 | 0.001 | 0.017 |
| CKB | DOWN | 0.34 | <0.001 | 0.002 |
| ZSCAN1 | DOWN | 0.33 | <0.001 | 0.011 |
| KCND3 | DOWN | 0.33 | <0.001 | 0.001 |
| COL25A1 | DOWN | 0.33 | <0.001 | <0.001 |
| WNK4 | DOWN | 0.33 | <0.001 | <0.001 |
| NTRK2 | DOWN | 0.32 | <0.001 | 0.003 |
| LOC107984400 | DOWN | 0.32 | <0.001 | <0.001 |
| LOC105375634 | DOWN | 0.32 | <0.001 | 0.003 |
| LOC105369869 | DOWN | 0.32 | <0.001 | 0.001 |
| ELOVL2 | DOWN | 0.31 | <0.001 | <0.001 |
| SCN7A | DOWN | 0.30 | <0.001 | <0.001 |
| WDR11 | DOWN | 0.30 | <0.001 | 0.001 |
| LOC102724484 | DOWN | 0.30 | <0.001 | 0.001 |
| MACROD2 | DOWN | 0.30 | <0.001 | <0.001 |
| LOC105372877 | DOWN | 0.29 | <0.001 | <0.001 |
| STC2 | DOWN | 0.29 | <0.001 | 0.007 |
| NAV3 | DOWN | 0.29 | <0.001 | 0.002 |
| LIN7A | DOWN | 0.29 | <0.001 | <0.001 |
| MACROD2-AS1 | DOWN | 0.28 | <0.001 | <0.001 |
| SORCS1 | DOWN | 0.28 | <0.001 | 0.005 |
| LOC101926959 | DOWN | 0.27 | <0.001 | 0.001 |
| FYB2 | DOWN | 0.27 | <0.001 | <0.001 |
| IGF2 | DOWN | 0.27 | <0.001 | <0.001 |
| IL33 | DOWN | 0.27 | 0.001 | 0.012 |
| KRT14 | DOWN | 0.27 | 0.002 | 0.028 |
| RBM24 | DOWN | 0.27 | 0.001 | 0.015 |
| NTRK3 | DOWN | 0.26 | <0.001 | 0.004 |
| PGR | DOWN | 0.25 | 0.002 | 0.027 |
| CNR1 | DOWN | 0.25 | <0.001 | 0.003 |
| PIEZO2 | DOWN | 0.25 | 0.001 | 0.017 |
| LOC105375886 | DOWN | 0.23 | <0.001 | 0.002 |
| ERICH3 | DOWN | 0.22 | <0.001 | 0.002 |
| DPY19L2P4 | DOWN | 0.22 | <0.001 | 0.006 |
| NECAB1 | DOWN | 0.20 | 0.004 | 0.043 |
| ADH1B | DOWN | 0.19 | <0.001 | <0.001 |
| PI15 | DOWN | 0.17 | <0.001 | <0.001 |
| FGFR2 | DOWN | 0.16 | <0.001 | 0.004 |
| GRIA2 | DOWN | 0.15 | <0.001 | 0.005 |
| LOC105375472 | DOWN | 0.13 | <0.001 | <0.001 |
| PCSK1 | DOWN | 0.04 | <0.001 | 0.005 |

**Table S2.** List of the 135 differentially regulated genes in the SUV-H *vs*. SUV-L BCs of the 120-PET cohort. The statistical analysis of the SUV-H and SUV-L groups was performed using the EdgeR package of R software (version 3.40.2). p-values and false discovery rates (FDR) were obtained using quasi-likelihood F-tests (QLF) and further adjusted using the Benjamini-Hochberg correction procedure. UP, upregulated genes (FDR < 0.05, p < 0.05, FC > 2.5, 73 genes); DOWN, downregulated genes (FDR < 0.05, p < 0.05, FC < 0.4, 62 genes). FC, fold-change SUV-H *vs.* SUV-L.

**Table S3. Functions of the 135 genes differentially expressed between SUV-H *vs.* SUV-L BCs of the 120-PET cohort.**

| **Category/**  **Function** | **Upregulated Genes**  **(PETsign genes are in bold)** | **Downregulated Genes**  **(PETsign genes are in bold)** |
| --- | --- | --- |
| ncRNA  (11 UP, 17 DOWN) | CCDC144NL-AS1, LINC00673, LOC105371822, LOC105374557, LOC105375170, LOC105375172, LOC105376442, LOC107986350, LOC107987296, LOC112268106, TGFB2-OT1. | DPY19L2P4, LINC01016, LOC101929322, LOC101926959, LOC102724484, LOC105369869, LOC105372877, LOC105374020, LOC105375387, LOC105375472, LOC105375634, LOC105375886, LOC105375896, LOC105378747, LOC107984400, MACROD2-AS1, ME3-DT. |
| Immune/inflammation-related (15 UP, 2 DOWN) | **ADM**, **CSF2RB**, **CXCL8**, **CXCL10**, **CXCL11**, CXCL13, CHI3L1, HERC5, KLHDC7B, **LAMP3**, NLRP2, **IFI27**, IGHG4, **S100A9**, **SPP1**. | **IL33**, **FREM1**. |
| Ion transport at the PM, other transporters and/or carrier (10 UP, 11 DOWN) | **AQP9**, ATP8A2, **CP**, **GABRE**, GABRP, KCNJ6, **MFSD2A**, **SEC61G**, SLC28A3, SLC34A2. | **ABCA10**, **FOLH1**, GRIA2, GRIK4, KCND3, LIN7A, **PIEZO2**, SCN7A, SLC22A3, **STC2**, **WNK4**. |
| Signal transduction, DNA replication/mitosis, traffic (13 UP, 13 DOWN) | **CDCA7**, **CENPW**, **EGFR**, EREG, MAB21L4, **MCM10**, PAK3, PDE4B, **PLCH1**, **SHISA2**, **SLITRK5**, TFPI2, **TMEM158**. | CNR1, FGFR2, **FYB2**, IGF2, **MACROD2**, NECAB1, **NTRK2**, NTRK3, PCSK1, PDE11A, **PGR**, **SORCS1**, WDR11. |
| Metabolism, mitochondrial function, modifying enzymes (14 UP, 6 DOWN) | B3GNT5, **C5orf46**, CYP1B1, **GALNT14**, HPGD, **ME1**, MOXD1, **NDUFA4L2**, PRR16, **PSAT1**, **RARRES1**, SPTSSB, THRSP, UBD. | ADH1B, **CKB**, **CYP2A7**, **ELOVL2**, **PI15**, **TMEM26**. |
| Cell adhesion, shape and/or motility, including ciliary proteins (6 UP, 11 DOWN) | **ADAMDEC1**, **CDH3**, CLDN1, CLDN16, DNAH11, MMP1. | ADIPOQ, CCDC158, **CCDC85A**, COL25A1, **ERICH3**, **KRT14**, KRT15, **NAV3**, **NOVA1**, NRCAM, RELN. |
| Regulators of gene expression (4 UP, 2 DOWN) | EPOP, **PIR**, SIM1, **SOX11**. | RBM24, ZSCAN1. |

**Table S3.** The functional categories were attributed manually by interrogating the NCBI “Gene” database, followed by inspection of extant literature. Genes of the 54-gene PET signature (PETsign) are in bold.

Evidence in literature indicates an involvement of most of the 54 PETsign genes in BC. This evidence was obtained at various levels of resolution and strength by *in vitro* genetic manipulation of cell lines or analysis of clinical tumor samples and/or bodily fluids from BC patients. In nearly all cases, the direction of gene regulation (UP or DOWN), as detected by PETsign, corresponds with the enhancement or reduction of parameters of aggressiveness, respectively. Below, we provide a list of some of the papers that analyze individual genes (or small groups of genes) from the PETsign, with no claim to comprehensiveness.

UP genes: ADM,^[3]^ AQP9,^[4]^ CDCA7,^[5]^ CDH3,^[6]^ CENPW,^[7]^ CP,^[8]^ CSF2RB,^[9]^ CXCL10,^[10]^ CXCL11,^[10a, 11]^ CXCL8,^[12]^ EGFR,^[13]^ (as an example from a wealth of papers on the subject), GALNT14,^[14]^ LAMP3,^[15]^ MCM10,^[16]^ ME1,^[17]^ NDUFA4L2,^[18]^ PIR,^[19]^ PSAT1,^[20]^ RARRES1,^[21]^ S100A9,^[22]^ SEC61G,^[23]^ SOX11,^[24]^ SPP1.^[25]^

DOWN genes: ABCA10,^[26]^ ELOVL2,^[27]^ FOLH1,^[28]^ FREM1,^[29]^ NAV3,^[30]^ PIEZO2,^[31]^ STC2.^[32]^

**Table S4: The immune-related genes of PETsign are not a reflection of different immune infiltrates.**

| **Gene** | **Cell Type** | **FC SUV-H/SUV-L** | **p-value** | **FDR** |
| --- | --- | --- | --- | --- |
| BLK | B cell | ND |  |  |
| CD19 | B cell | ND |  |  |
| MS4A1 | B cell | 0.68 | 0.17 | 0.45 |
| TNFRSF17 | B cell | ND |  |  |
| PTPRC | CD45 | 1.17 | 0.26 | 0.57 |
| CD8A | CD8 T cell | 0.93 | 0.66 | 0.87 |
| CD8B | CD8 T cell | 0.80 | 0.24 | 0.55 |
| CTSW | Cytotoxic cell | ND |  |  |
| GNLY | Cytotoxic cell | ND |  |  |
| GZMA | Cytotoxic cell | ND |  |  |
| GZMB | Cytotoxic cell | ND |  |  |
| GZMH | Cytotoxic cell | ND |  |  |
| KLRB1 | Cytotoxic cell | 1.01 | 0.91 | 0.97 |
| KLRD1 | Cytotoxic cell | 0.93 | 0.10 | 0.34 |
| KLRK1 | Cytotoxic cell | 0.76 | 0.14 | 0.42 |
| PRF1 | Cytotoxic cell | ND |  |  |
| CCL13 | Dendritic cell | ND |  |  |
| CD209 | Dendritic cell | 0.99 | 0.95 | 0.98 |
| HSD11B1 | Dendritic cell | ND |  |  |
| CD244 | Exhausted CD8 T cell | ND |  |  |
| EOMES | Exhausted CD8 T cell | 0.98 | 0.91 | 0.97 |
| LAG3 | Exhausted CD8 T cell | ND |  |  |
| CD163 | Macrophage | 1.25 | 0.14 | 0.40 |
| CD68 | Macrophage | 1.37 | 0.10 | 0.34 |
| CD84 | Macrophage | 1.13 | 0.16 | 0.44 |
| MS4A2 | Mast cell | 0.84 | 0.37 | 0.67 |
| TPSAB1 | Mast cell | 0.47 | 0.008 | 0.071 |
| CSF3R | Neutrophil | 1.08 | 0.74 | 0.91 |
| FCGR3A | Neutrophil | 1.46 | 0.0021 | 0.030 |
| S100A12 | Neutrophil | ND |  |  |
| IL21R | Natural killer CD56^dim^ cell | 1.27 | 0.18 | 0.47 |
| KIR3DL1 | Natural killer CD56^dim^ cell | ND |  |  |
| KIR3DL2 | Natural killer CD56^dim^ cell | ND |  |  |
| KIR3DL3 | Natural killer CD56^dim^ cell | ND |  |  |
| NCR1 | Natural killer cell | ND |  |  |
| XCL2 | Natural killer cell | ND |  |  |
| CD3D | T cell | ND |  |  |
| CD3E | T cell | 0.89 | 0.54 | 0.80 |
| CD3G | T cell | 1.06 | 0.65 | 0.87 |
| CD6 | T cell | 0.79 | 0.26 | 0.57 |
| SH2D1A | T cell | 1.17 | 0.46 | 0.75 |
| TBX21 | Type 1 T helper cell | ND |  |  |
| FOXP3 | Regulatory T cell | 0.98 | 0.91 | 0.97 |

**Table S4.** Since several of the upregulated genes in SUV-H BCs are annotated as immune/inflammation-related, we performed several controls to establish whether their presence was due to increased immune infiltration in SUV-H tumors. The results shown in this table were generated starting from the “nCounter Human Pan-Cancer Immune Profiling Panel; NanoString Technologies” which is widely used to establish immune components in a tumor mass (see for instance,^[33]^). From the genes listed in the panel, we extracted the genes labeled as “cell-specific” as they should specifically reflect the presence of immune cells. Several genes were not detected (ND) in BCs, regardless of their SUV status. With two exceptions (*TPSAB1* and *FCGR3A*), the detectable genes did not show significant differences between SUV-H and SUV-L BCs. In the case of *TPSAB1* and *FCGR3A*, it should be noted that other genes identifying the same immune cell type (mast cells and neutrophils, respectively) were not differentially expressed in the SUV-H *vs*. SUV-L comparison.

We also performed additional analyses using TIMER 2.0,^[34]^ which revealed minimal contamination of immune/stromal components in the analyzed BC samples (see Table S5). We concluded that the presence of transcripts belonging to genes annotated as immune/inflammatory is due to the epithelial component of the analyzed BCs.

Statistical analysis was performed using the EdgeR package in R software (version 3.40.2) to calculate fold-change (FC), p-values and false discovery rates (FDRs); please refer to the legend to Table S2 and the section on Data Analysis and Statistical Methods for further details.

**Table S5: Analysis of immune infiltrates and stromal contamination in the analyzed BCs.**

| **Cell Type** | **Algorithm** | **Median SUV-H BCs** | **Median SUV-L BCs** |
| --- | --- | --- | --- |
| B cell memory | CIBERSORT-ABS | 0.01 | 0.02 |
| B cell naïve | CIBERSORT-ABS | 0.00 | 0.00 |
| B cell plasma | CIBERSORT-ABS | 0.03 | 0.03 |
| Eosinophil | CIBERSORT-ABS | 0.00 | 0.00 |
| Macrophage M0 | CIBERSORT-ABS | 0.00 | 0.00 |
| Macrophage M1 | CIBERSORT-ABS | 0.06 | 0.03 |
| Macrophage M2 | CIBERSORT-ABS | 0.20 | 0.19 |
| Mast cell activated | CIBERSORT-ABS | 0.04 | 0.04 |
| Mast cell resting | CIBERSORT-ABS | 0.00 | 0.00 |
| Monocyte | CIBERSORT-ABS | 0.01 | 0.01 |
| Myeloid dendritic cell activated | CIBERSORT-ABS | 0.04 | 0.03 |
| Myeloid dendritic cell resting | CIBERSORT-ABS | 0.00 | 0.00 |
| Neutrophil | CIBERSORT-ABS | 0.00 | 0.00 |
| NK cell activated | CIBERSORT-ABS | 0.01 | 0.01 |
| NK cell resting | CIBERSORT-ABS | 0.00 | 0.00 |
| T cell CD4+ memory activated | CIBERSORT-ABS | 0.00 | 0.00 |
| T cell CD4+ memory resting | CIBERSORT-ABS | 0.18 | 0.13 |
| T cell CD4+ naïve | CIBERSORT-ABS | 0.00 | 0.00 |
| T cell CD8+ | CIBERSORT-ABS | 0.03 | 0.04 |
| T cell follicular helper | CIBERSORT-ABS | 0.04 | 0.02 |
| T cell gamma delta | CIBERSORT-ABS | 0.00 | 0.00 |
| T cell regulatory (Tregs) | CIBERSORT-ABS | 0.00 | 0.00 |
| B cell | EPIC | 0.00 | 0.00 |
| Cancer associated fibroblast | EPIC | 0.06 | 0.08 |
| Endothelial cell | EPIC | 0.01 | 0.01 |
| Macrophage | EPIC | 0.00 | 0.00 |
| NK cell | EPIC | 0.00 | 0.00 |
| T cell CD4+ | EPIC | 0.05 | 0.04 |
| T cell CD8+ | EPIC | 0.01 | 0.01 |
| Uncharacterized cell | EPIC | 0.85 | 0.83 |
| B cell | QUANTISEQ | 0.01 | 0.01 |
| Macrophage M1 | QUANTISEQ | 0.01 | 0.00 |
| Macrophage M2 | QUANTISEQ | 0.01 | 0.01 |
| Monocyte | QUANTISEQ | 0.00 | 0.00 |
| Myeloid dendritic cell | QUANTISEQ | 0.00 | 0.00 |
| Neutrophil | QUANTISEQ | 0.01 | 0.01 |
| NK cell | QUANTISEQ | 0.01 | 0.01 |
| T cell CD4+ (non-regulatory) | QUANTISEQ | 0.00 | 0.00 |
| T cell CD8+ | QUANTISEQ | 0.00 | 0.00 |
| T cell regulatory (Tregs) | QUANTISEQ | 0.01 | 0.01 |
| Uncharacterized cell | QUANTISEQ | 0.95 | 0.95 |

**Table S5.** We used the TIMER 2.0 webtool,^[34]^ which integrates multiple state-of-the-art algorithms for the estimation of immune and non-immune cell infiltration on user-provided expression profiles, to compare SUV-H and SUV-L BCs of the 120-BC PET cohort. We report here the data obtained with the EPIC,^[35]^ and QUANTISEQ^[36]^ algorithms that provide absolute scores representing cell fractions: the numbers in the table correspond to percentage of cells reported as a decimal. Additionally, we report the data obtained with the CYBERSORT algorithm in the absolute mode (CYBERSORT-ABS),^[37]^ which scales cellular fractions to a score (in arbitrary units) that reflects each cell type’s absolute proportion, allowing comparison across both samples and cell types. The data show that the contamination by immune, endothelial and fibroblast components is minimal in the analyzed BCs, with most of the cells (as evidenced by the EPIC and QUANTISEQ algorithms) classified as “Uncharacterized cell” (in bold), presumably epithelial cells.

**Table S6. Characteristics of the METABRIC, TCGA AND 970-IEO clinical cohorts used for the derivation and validation of PETsign.**

| **Cohort** | **Cases (N)** | **Clinical and Pathological Parameters** | **Endpoint** | **Average**  **Follow-up** |
| --- | --- | --- | --- | --- |
| METABRIC * | 1904 | Age, TNM, HR (ER/PGR), HER2, Grade | DRBC | 10.21 years |
| TCGA ** | 896 | Age, TNM, HR (ER/PGR), HER2 | OS | 3.58 years |
| 970-IEO *** | 970 | Age, TNM, HR (ER/PGR), HER2, Grade, Ki-67 | DRBC | 12.45 years |

**Table S6.** The three BC cohorts utilized in this study are shown. The METABRIC cohort corresponds to all 1904 cases present in the dataset and downloadable from the cBioPortal. There is no information in this cohort on the M0 or M1 status of patients at diagnosis. The Cancer Genome Atlas (TCGA) cohort consists of the 896 M0 cases, from the 1108 total cases available in the TCGA Firehose Legacy dataset and downloadable from the cBioPortal. The 970-IEO cohort comprises 970 M0 patients selected from the larger consecutive IEO cohort described in Table S8. For all cohorts, the table shows the clinicopathological parameters available for multivariable analysis and the endpoints available for prognostic analysis: death related to BC (DRBC); overall survival (OS).

* The METABRIC case collection was assembled from tumor banks in the UK and Canada.^[38]^ RNA was extracted from fresh-frozen breast tissue samples. Transcriptomic analysis was performed using the Illumina HumanHT-12 v3.0 Gene Expression BeadChip.

** TCGA was a joint effort of the National Cancer Institute (NCI) and the National Human Genome Research Institute (NHGRI), which are both part of the National Institutes of Health, U.S. Department of Health and Human Services.^[39]^ Tissue samples were accrued from a number of institutions, mainly based in the US, but also in Russia, Poland and Vietnam. For breast samples, RNA was extracted from fresh-frozen tissues. Transcriptomic analysis was performed using the Illumina HiSeq 2000 System.

*** The IEO cohort is a single institution consecutive cohort collected at the European Institute of Oncology (IEO) in Milan, Italy, between the years 1997 and 2000.^[40]^ The 970-patient sub-cohort was matched for clinicopathological characteristics to the entire cohort (see Table S8). RNAseq was performed on the sub-cohort for the purpose of the present study. RNA was extracted from FFPE specimens, as detailed in the main text. Transcriptomic analysis was performed using the Novaseq 6000 sequencer.

**Table S7. Derivation of the 54-gene prognostic PETsign by interrogation of the METABRIC dataset.**

| **Gene Name** | **Reg.** | **Approved Name** | **HR** | **P** |
| --- | --- | --- | --- | --- |
| ADAMDEC1 | UP | ADAM-like decysin 1 | 1.36 | <0.001 |
| ADM | UP | Adrenomedullin | 1.48 | <0.001 |
| AQP9 | UP | Aquaporin 9 | 1.71 | <0.001 |
| C5orf46 | UP | Chromosome 5 open reading frame 46 | 1.22 | 0.024 |
| CDCA7 | UP | Cell division cycle associated 7 | 1.64 | <0.001 |
| CDH3 | UP | Cadherin 3 | 1.29 | 0.004 |
| CENPW | UP | Centromere protein W | 1.42 | <0.001 |
| CP | UP | Ceruloplasmin | 1.43 | <0.001 |
| CSF2RB | UP | Colony stimulating factor 2 receptor subunit beta | 1.22 | 0.012 |
| CXCL10 | UP | C-X-C motif chemokine ligand 10 | 1.43 | <0.001 |
| CXCL11 | UP | C-X-C motif chemokine ligand 11 | 1.24 | 0.020 |
| CXCL8 | UP | C-X-C motif chemokine ligand 8 | 1.55 | <0.001 |
| EGFR | UP | Epidermal growth factor receptor | 1.55 | <0.001 |
| GABRE | UP | Gamma-aminobutyric acid type A receptor subunit epsilon | 1.21 | 0.040 |
| GALNT14 | UP | Polypeptide N-acetylgalactosaminyltransferase 14 | 1.29 | 0.002 |
| IFI27 | UP | Interferon alpha-inducible protein 27 | 1.27 | 0.004 |
| LAMP3 | UP | Lysosomal associated membrane protein 3 | 1.26 | 0.009 |
| MCM10 | UP | Minichromosome maintenance 10 replication initiation factor | 1.88 | <0.001 |
| ME1 | UP | Malic enzyme 1 | 1.30 | 0.002 |
| MFSD2A | UP | Major facilitator superfamily domain containing 2A | 1.43 | <0.001 |
| NDUFA4L2 | UP | NDUFA4 mitochondrial complex associated like 2 | 1.30 | 0.006 |
| PIR | UP | Pirin | 1.36 | <0.001 |
| PLCH1 | UP | Phospholipase C eta-1 | 1.34 | <0.001 |
| PSAT1 | UP | Phosphoserine aminotransferase 1 | 1.49 | <0.001 |
| RARRES1 | UP | Retinoic acid receptor responder 1 | 1.29 | 0.008 |
| S100A9 | UP | S100 calcium binding protein A9 | 1.56 | <0.001 |
| SEC61G | UP | SEC61 translocon subunit gamma | 1.29 | 0.002 |
| SHISA2 | UP | Shisa family member 2 | 0.83 | 0.048 |
| SLITRK5 | UP | SLIT and NTRK like family member 5 | 1.27 | 0.006 |
| SOX11 | UP | SRY-box transcription factor 11 | 1.80 | <0.001 |
| SPP1 | UP | Secreted phosphoprotein 1 | 1.37 | <0.001 |
| TMEM158 | UP | Transmembrane protein 158 | 1.27 | 0.007 |
| ABCA10 | DOWN | ATP binding cassette subfamily A member 10 | 0.74 | <0.001 |
| CCDC85A | DOWN | Coiled-coil domain containing 85A | 0.66 | <0.001 |
| CKB | DOWN | Creatine kinase B | 1.44 | <0.001 |
| CYP2A7 | DOWN | Cytochrome P450 family 2 subfamily A member 7 | 0.61 | 0.013 |
| ELOVL2 | DOWN | ELOVL fatty acid elongase 2 | 0.67 | <0.001 |
| ERICH3 | DOWN | Glutamate rich 3 | 0.54 | <0.001 |
| FOLH1 | DOWN | Folate hydrolase 1 | 1.34 | <0.001 |
| FREM1 | DOWN | FRAS1-related extracellular matrix 1 | 0.84 | 0.034 |
| FYB2 | DOWN | FYN binding protein 2 | 0.79 | 0.009 |
| IL33 | DOWN | Interleukin 33 | 0.72 | <0.001 |
| KRT14 | DOWN | Keratin 14 | 0.75 | 0.007 |
| MACROD2 | DOWN | Mono-ADP ribosylhydrolase 2 | 1.22 | 0.014 |
| NAV3 | DOWN | Neuron navigator 3 | 0.66 | 0.001 |
| NOVA1 | DOWN | NOVA alternative splicing regulator 1 | 0.67 | <0.001 |
| NTRK2 | DOWN | Neurotrophic receptor tyrosine kinase 2 | 0.65 | <0.001 |
| PGR | DOWN | Progesterone receptor | 0.59 | <0.001 |
| PI15 | DOWN | Peptidase inhibitor 15 | 0.71 | 0.006 |
| PIEZO2 | DOWN | Piezo type mechanosensitive ion channel component 2 | 0.61 | <0.001 |
| SORCS1 | DOWN | Sortilin-related VPS10 domain containing receptor 1 | 0.80 | 0.026 |
| STC2 | DOWN | Stanniocalcin 2 | 0.56 | <0.001 |
| TMEM26 | DOWN | Transmembrane protein 26 | 0.47 | <0.001 |
| WNK4 | DOWN | WNK lysine deficient protein kinase 4 | 0.77 | 0.004 |
| ATP8A2 | UP | Atpase phospholipid transporting 8A2 | 1.14 | 0.119 |
| B3GNT5 | UP | UDP-glcnac:betagal beta-1,3-N-acetylglucosaminyltransferase 5 | 1.07 | 0.388 |
| CLDN1 | UP | Claudin 1 | 1.16 | 0.102 |
| CXCL13 | UP | C-X-C motif chemokine ligand 13 | 0.87 | 0.180 |
| CYP1B1 | UP | Cytochrome P450 family 1 subfamily B member 1 | 0.91 | 0.310 |
| GABRP | UP | Gamma-aminobutyric acid type A receptor subunit pi | 1.16 | 0.180 |
| HPGD | UP | 15-hydroxyprostaglandin dehydrogenase | 0.89 | 0.355 |
| KCNJ6 | UP | Potassium inwardly rectifying channel subfamily J member 6 | 0.98 | 0.851 |
| KLHDC7B | UP | Kelch domain-containing 7B | 1.13 | 0.140 |
| CLDN16 | UP | Claudin 16 | 1.01 | 0.864 |
| MOXD1 | UP | Monooxygenase DBH-like 1 | 0.95 | 0.522 |
| MMP1 | UP | Matrix metallopeptidase 1 | 1.12 | 0.240 |
| NLRP2 | UP | NLR family pyrin domain-containing 2 | 1.01 | 0.881 |
| PAK3 | UP | P21 (RAC1) activated kinase 3 | 0.87 | 0.094 |
| PDE4B | UP | Phosphodiesterase 4B | 0.97 | 0.670 |
| PRR16 | UP | Proline rich 16 | 0.94 | 0.493 |
| SIM1 | UP | SIM bhlh transcription factor 1 | 1.15 | 0.092 |
| SLC28A3 | UP | Solute carrier family 28 member 3 | 0.89 | 0.247 |
| SLC34A2 | UP | Solute carrier family 34 member 2 | 1.00 | 0.974 |
| SPTSSB | UP | Serine palmitoyltransferase small subunit B | 1.03 | 0.772 |
| TFPI2 | UP | Tissue factor pathway inhibitor 2 | 1.03 | 0.808 |
| THRSP | UP | Thyroid hormone responsive | 0.86 | 0.130 |
| UBD | UP | Ubiquitin D | 0.91 | 0.290 |
| CCDC158 | DOWN | Coiled-coil domain containing 158 | 0.99 | 0.886 |
| CNR1 | DOWN | Cannabinoid receptor 1 | 1.09 | 0.384 |
| COL25A1 | DOWN | Collagen type XXV alpha 1 chain | 1.12 | 0.153 |
| DPY19L2P4 | DOWN | DPY19L2 pseudogene 4 | 0.94 | 0.526 |
| FGFR2 | DOWN | Fibroblast growth factor receptor 2 | 0.99 | 0.893 |
| GRIA2 | DOWN | Glutamate ionotropic receptor AMPA type subunit 2 | 0.86 | 0.170 |
| GRIK4 | DOWN | Glutamate ionotropic receptor kainate type subunit 4 | 0.88 | 0.122 |
| IGF2 | DOWN | Insulin-like growth factor 2 | 0.95 | 0.543 |
| KCND3 | DOWN | Potassium voltage-gated channel subfamily D member 3 | 1.09 | 0.324 |
| KRT15 | DOWN | Keratin 15 | 0.84 | 0.086 |
| LIN7A | DOWN | Lin-7 homolog A, crumbs cell polarity complex component | 1.16 | 0.096 |
| NECAB1 | DOWN | N-terminal EF-hand calcium binding protein 1 | 0.84 | 0.195 |
| NRCAM | DOWN | Neuronal cell adhesion molecule | 0.98 | 0.863 |
| NTRK3 | DOWN | Neurotrophic receptor tyrosine kinase 3 | 0.95 | 0.521 |
| PCSK1 | DOWN | Proprotein convertase subtilisin/kexin type 1 | 0.75 | 0.140 |
| PDE11A | DOWN | Phosphodiesterase 11A | 0.97 | 0.669 |
| RBM24 | DOWN | RNA binding motif protein 24 | 0.98 | 0.837 |
| RELN | DOWN | Reelin | 0.98 | 0.817 |
| SCN7A | DOWN | Sodium voltage-gated channel alpha subunit 7 | 0.97 | 0.738 |
| SLC22A3 | DOWN | Solute carrier family 22 member 3 | 1.08 | 0.360 |
| WDR11 | DOWN | WD repeat domain 11 | 0.85 | 0.060 |
| ZSCAN1 | DOWN | Zinc finger and SCAN domain-containing 1 | 1.06 | 0.498 |

**Table S7.** List of the 99 genes, from the 135 differentially expressed genes between SUV-H *vs.* SUV-L BCs, for which expression data were available in the METABRIC database. For each gene, the gene name, mode of regulation (Reg.: up or down in the 135 gene list, see Table S2), approved name *in extenso*, hazard ratio (HR) for death related to BC in univariate analysis, p-value (P) in univariate analysis. P-values and HR were calculated by Cox proportional hazards regression model analysis using the ‘survival’ package in R, version 3.5-5. The 54 genes significantly correlating with worse prognostic outcome in univariate analysis are shaded in grey. These genes comprise the PETsign.

We also calculated whether there was a significant enrichment of genes predicting prognosis in univariate analysis in the 99-gene list compared with the entire METABRIC dataset. From the 24,368 “genes” listed in the dataset, at the time of analysis, 6632 (27.22%) were predictive of prognosis in univariate analysis, compared to 54/99 in the PET list (54.55%), when a p of 0.05 was used. By adopting a more stringent p-value of 0.01, 4336 of 24,368 genes were predictive (17.38%) *vs*. 45 of 99 (45.45%) genes from the PET list. This represents a 2-fold and 2.6-fold increase in prognostic genes in the PET *vs.* METABRIC gene list, respectively. In both cases, the difference in the frequency of prognostic genes between the two gene lists was highly significant: P < 0.00001 by Fischer’s exact test.

**Table S8. Prognostic stratification by PETsign in the METABRIC, TCGA, and 970-IEO clinical cohorts.**

| **Cohort** | **Subgroup and Type of Analysis** | **HR** | **P** | **95% CI** |
| --- | --- | --- | --- | --- |
| METABRIC | ALL Univariate | 2.09 | <0.0001 | 1.77-2.45 |
|  | ALL Multivariable | 1.62 | <0.0001 | 1.33-1.98 |
|  | LUMINAL Univariate | 1.79 | <0.0001 | 1.47-2.18 |
|  | LUMINAL Multivariable | 1.57 | <0.0001 | 1.28-1.92 |
|  | LUMINAL-HER2 Univariate | 1.24 | 0.49 | 0.67-2.29 |
|  | HER2 Univariate | 1.18 | 0.57 | 0.66-2.11 |
|  | TNBC Univariate | 0.97 | 0.86 | 0.67-1.40 |
| TCGA* | ALL Univariate | 2.32 | 0.0004 | 1.46-3.71 |
|  | ALL Multivariable | 2.18 | 0.0059 | 1.25-3.78 |
| 970-IEO | ALL Univariate | 2.74 | <.0001 | 1.99-3.78 |
|  | ALL Multivariable | 2.27 | <.0001 | 1.56-3.32 |
|  | LUMINAL-A Univariate | Classes not assigned** | | |
|  | LUMINAL-B Univariate | 2.38 | <0.0001 | 1.56-3.62 |
|  | LUMINAL-B Multivariable | 1.66 | 0.026 | 1.06-2.58 |
|  | LUMINAL (A+B) Univariate | 1.82 | <0.0001 | 1.49-2.22 |
|  | LUMINAL (A+B) Multivariable | 1.60 | <0.0001 | 1.30-1.96 |
|  | LUM-HER2 Univariate | 1.91 | 0.22 | 0.68-5.37 |
|  | HER2 Univariate | Classes not assigned*** | | |
|  | TNBC Univariate | 1.25 | 0.71 | 0.39-3.97 |

**Table S8.** The prognostic performance of PETsign in the three clinical cohorts is shown. Hazard ratios (HR) and p-values (P) were calculated with the Cox proportional hazards model using JMP. The 95% confidence intervals (CI) are also shown. Significant values are indicated in red. Variables used for the multivariable analyses were as follows. METABRIC: age, tumor size, nodal status, HR (ER/PGR), HER2, and tumor grade. TCGA: age, tumor size, nodal status, HR (ER/PGR), and HER2. IEO: age, tumor size, nodal status, HR (ER/PGR), HER2, tumor grade and Ki67 status.

*, In the TCGA dataset, at 5 years of follow-up, there were only 63 events (deaths). This prevented meaningful analysis of the BC molecular subgroups, since too few events were present (26 for Luminal, 12 for Luminal-HER2, 4 for HER2, 21 for TNBC).

**, Classes could not be assigned because only 4 events (DRBC) were present, all SUV-H.

***, Hierarchical clustering applied to HER2-positive samples did not result in two classes of tumors, resembling SUV-H-like or SUV-L-like, because almost all tumors were SUV-H-like.

**Table S9. Clinicopathological characteristics of the complete IEO cohort and the 970-patient sub-cohort.**

|  | **Complete Cohort** | | **970-Patient Sub-cohort** | |  |
| --- | --- | --- | --- | --- | --- |
| **Category** | **Number** | **%** | **Number** | **%** | **χ2 p-Value** |
| Age <50 | 911 | 39.34% | 368 | 37.94% |  |
| Age ≥50 | 1405 | 60.67% | 602 | 62.06% |  |
| Age Total | 2316 | 100.00% | 970 | 100.00% | 0.45 |
| T1 | 1493 | 64.47% | 616 | 63.51% |  |
| T2 | 744 | 32.12% | 322 | 33.20% |  |
| T3 | 61 | 2.63% | 22 | 2.27% |  |
| T4 | 18 | 0.78% | 10 | 1.03% |  |
| T Total | 2316 | 100.00% | 970 | 100.00% | 0.75 |
| pN NEG | 1124 | 49.71% | 462 | 48.63% |  |
| pN POS | 1137 | 50.29% | 488 | 51.37% |  |
| pN Total | 2261 | 100.00% | 950 | 100.00% | 0.74 |
| pN Missing | 55 |  | 20 |  |  |
| Grade G1 | 427 | 18.88% | 182 | 19.16% |  |
| Grade G2 | 1009 | 44.61% | 414 | 43.58% |  |
| Grade G3 | 826 | 36.52% | 354 | 37.26% |  |
| Grade Total | 2262 | 100.00% | 950 | 100.00% | 0.92 |
| Grade Missing | 54 |  | 20 |  |  |
| PGR NEG | 647 | 27.94% | 281 | 28.97% |  |
| PGR POS | 1669 | 72.06% | 689 | 71.03% |  |
| PGR Total | 2316 | 100.00% | 970 | 100.00% | 0.55 |
| ER NEG | 345 | 14.90% | 146 | 15.05% |  |
| ER POS | 1971 | 85.10% | 824 | 84.95% |  |
| ER Total | 2316 | 100.00% | 970 | 100.00% | 0.91 |
| Ki67 <14% | 653 | 28.22% | 279 | 28.79% |  |
| Ki67 >14% | 1661 | 71.78% | 690 | 71.21% |  |
| Ki67 Total | 2314 | 100.00% | 969 | 100.00% | 0.74 |
| Ki67 Missing | 2 |  | 2 |  |  |
| HER2 (IHC) NEG | 1826 | 87.83% | 790 | 87.58% |  |
| HER2 (IHC) POS | 253 | 12.17% | 112 | 12.42% |  |
| HER2 (IHC) Total | 2079 | 100.00% | 902 | 100.00% | 0.85 |
| HER2 (IHC) Missing | 237 |  | 68 |  |  |
| LUMINAL A | 432 | 18.82% | 184 | 19.05% |  |
| LUMINAL B | 1414 | 61.59% | 587 | 60.77% |  |
| LUMINAL B HER2 POS | 152 | 6.62% | 66 | 6.83% |  |
| HER2 | 101 | 4.40% | 46 | 4.76% |  |
| TNBC | 197 | 8.58% | 83 | 8.59% |  |
| Subtype Total | 2296 | 100.00% | 966 | 100.00% | 0.99 |
| Subtype Missing | 20 |  | 4 |  |  |
| Event Distant | 346 | 14.94% | 150 | 15.46% |  |
| Event Locoregional | 182 | 7.86% | 81 | 8.35% |  |
| No Event | 1421 | 61.36% | 567 | 58.45% |  |
| Other | 367 | 15.85% | 172 | 17.73% |  |
| Event Total | 2316 | 100.00% | 970 | 100.00% | 0.43 |
| Death Other | 44 | 1.90% | 23 | 2.37% |  |
| Death Other Tumor | 49 | 2.12% | 26 | 2.68% |  |
| Death ictus | 28 | 1.21% | 13 | 1.34% |  |
| Death Primary Tumor | 351 | 15.16% | 154 | 15.88% |  |
| Alive | 1844 | 79.62% | 754 | 77.73% |  |
| Total | 2316 | 100.00% | 970 | 100.00% | 0.67 |

**Table S9.** The clinicopathological characteristics of the complete IEO cohort (N=2316) and the 970-patient IEO sub-cohort are reported. The complete IEO Cohort has been previously described.^[40]^ Transcriptomic profiling by RNAseq was performed on the 970-patient subcohort. Statistical analysis (chi-square tests with Excel) shows that the subcohort is matched to the entire consecutive cohort.

**Table S10. Co-occurrence of PETsign and 135-signature gene amplification in the TGCA cohort.**

| **A** | **B** | **Neither** | **A Not B** | **B Not A** | **Both** | **Log2 Odds Ratio** | **q-Value** | **Tendency** |
| --- | --- | --- | --- | --- | --- | --- | --- | --- |
| **LAMP3** | **B3GNT5** | 843 | 0 | 1 | 35 | >3 | **<0.001** | Co-occurrence |
| **CLDN1** | **CLDN16** | 850 | 0 | 1 | 28 | >3 | **<0.001** | Co-occurrence |
| **CXCL10** | **CXCL11** | 859 | 0 | 0 | 20 | >3 | **<0.001** | Co-occurrence |
| **RARRES1** | **SPTSSB** | 851 | 1 | 5 | 22 | >3 | **<0.001** | Co-occurrence |
| **LAMP3** | **CLDN1** | 840 | 11 | 4 | 24 | >3 | **<0.001** | Co-occurrence |
| **B3GNT5** | **CLDN1** | 839 | 12 | 4 | 24 | >3 | **<0.001** | Co-occurrence |
| **LAMP3** | **CLDN16** | 839 | 11 | 5 | 24 | >3 | **<0.001** | Co-occurrence |
| **B3GNT5** | **CLDN16** | 838 | 12 | 5 | 24 | >3 | **<0.001** | Co-occurrence |
| **CXCL8** | **CXCL10** | 856 | 3 | 2 | 18 | >3 | **<0.001** | Co-occurrence |
| **CXCL8** | **CXCL11** | 856 | 3 | 2 | 18 | >3 | **<0.001** | Co-occurrence |
| **RARRES1** | **PLCH1** | 853 | 4 | 3 | 19 | >3 | **<0.001** | Co-occurrence |
| **CP** | **PLCH1** | 853 | 4 | 4 | 18 | >3 | **<0.001** | Co-occurrence |
| **PLCH1** | **SPTSSB** | 849 | 3 | 8 | 19 | >3 | **<0.001** | Co-occurrence |
| **S100A9** | **CHI3L1** | 722 | 52 | 49 | 56 | >3 | **<0.001** | Co-occurrence |
| **EREG** | **CXCL10** | 857 | 2 | 4 | 16 | >3 | **<0.001** | Co-occurrence |
| **EREG** | **CXCL11** | 857 | 2 | 4 | 16 | >3 | **<0.001** | Co-occurrence |
| **CXCL8** | **EREG** | 856 | 5 | 2 | 16 | >3 | **<0.001** | Co-occurrence |
| **RARRES1** | **CP** | 850 | 7 | 6 | 16 | >3 | **<0.001** | Co-occurrence |
| **CP** | **SPTSSB** | 846 | 6 | 11 | 16 | >3 | **<0.001** | Co-occurrence |
| **EGFR** | **SEC61G** | 859 | 8 | 0 | 12 | >3 | **<0.001** | Co-occurrence |
| **CXCL10** | **CXCL13** | 857 | 8 | 2 | 12 | >3 | **<0.001** | Co-occurrence |
| **CXCL11** | **CXCL13** | 857 | 8 | 2 | 12 | >3 | **<0.001** | Co-occurrence |
| **CXCL8** | **CXCL13** | 855 | 10 | 3 | 11 | >3 | **<0.001** | Co-occurrence |
| **EREG** | **CXCL13** | 857 | 8 | 4 | 10 | >3 | **<0.001** | Co-occurrence |
| **CLDN1** | **RARRES1** | 840 | 16 | 11 | 12 | >3 | **<0.001** | Co-occurrence |
| **CLDN16** | **RARRES1** | 839 | 17 | 11 | 12 | >3 | **<0.001** | Co-occurrence |
| **LAMP3** | **SPTSSB** | 830 | 22 | 14 | 13 | >3 | **<0.001** | Co-occurrence |
| **CLDN1** | **SPTSSB** | 836 | 16 | 15 | 12 | >3 | **<0.001** | Co-occurrence |
| **LAMP3** | **CP** | 834 | 23 | 10 | 12 | >3 | **<0.001** | Co-occurrence |
| **B3GNT5** | **SPTSSB** | 829 | 23 | 14 | 13 | >3 | **<0.001** | Co-occurrence |
| **B3GNT5** | **CP** | 833 | 24 | 10 | 12 | >3 | **<0.001** | Co-occurrence |
| **CLDN16** | **SPTSSB** | 835 | 17 | 15 | 12 | >3 | **<0.001** | Co-occurrence |
| **CLDN1** | **CP** | 840 | 17 | 11 | 11 | >3 | **<0.001** | Co-occurrence |
| **CLDN1** | **PLCH1** | 840 | 17 | 11 | 11 | >3 | **<0.001** | Co-occurrence |
| **CLDN16** | **CP** | 839 | 18 | 11 | 11 | >3 | **<0.001** | Co-occurrence |
| **CLDN16** | **PLCH1** | 839 | 18 | 11 | 11 | >3 | **<0.001** | Co-occurrence |
| **LAMP3** | **RARRES1** | 832 | 24 | 12 | 11 | >3 | **<0.001** | Co-occurrence |
| **B3GNT5** | **RARRES1** | 831 | 25 | 12 | 11 | >3 | **<0.001** | Co-occurrence |
| **LAMP3** | **PLCH1** | 832 | 25 | 12 | 10 | >3 | **<0.001** | Co-occurrence |
| **B3GNT5** | **PLCH1** | 831 | 26 | 12 | 10 | >3 | **<0.001** | Co-occurrence |
| **MCM10** | **S100A9** | 755 | 16 | 94 | 14 | 2.813 | **<0.001** | Co-occurrence |

**Table S10.** The TCGA database was analyzed through the cBioPortal. Initially, the putative copy number alterations were evaluated by GISTIC 2.0 and only genes displaying amplification in at least 1.5% of cases were further considered. For these genes, the “mutual exclusivity function” of the cBioPortal was used to yield the numerical values shown in the Table. Only instances of highly significant co-occurrence (q-value < 0.001) are shown. In red, PETsign genes; in black, other upregulated genes from the list of 135 differentially expressed genes. In the table: columns A and B show the identity of gene A and gene B used in the pairwise comparison; Neither, number of samples displaying no amplification of A nor B; A not B, number of samples showing amplification of A but not B; B not A, number of samples exhibiting amplification of B but not A; Both, number of samples showing amplification of both A and B; Log2 Odds Ratio, log2(odds of amplification of B given amplification of A)/(odds of amplification of B given lack of amplification of A); q-value, derived from the two-sided Fisher’s Exact Test and the Benjamini-Hochberg FDR correction procedure; Tendency; result of the analysis showing highly significant co-occurrence of amplification of A and B.

Two genes, CCDC158 and ADIPOQ, were not included in our analysis, despite showing amplification in > 1.5% of BCs and significant co-occurrence with the other selected genes. This is because these two genes were downregulated in the PETsign. Since CCDC158 and ADIPOQ are located on chromosome 4q21.1 and 3q27.3, respectively, they are probably co-amplified in the amplicons reported in Figure 4c and 4e of the main text.

**Table S11: Metabolite production in SUV-H-like and SUV-L-like BC cell lines.**

| **Metabolite** | **FC SUV-H/SUV-L** | **p-value** |
| --- | --- | --- |
| 1-methylnicotinamide | 87.18 | <.0001 |
| GABA | 6.48 | 0.0023 |
| N-carbamoyl-beta-alanine | 3.65 | <.0001 |
| Kynurenine | 1.96 | 0.0371 |
| Inositol | 1.73 | 0.0001 |
| UDP-galactose/UDP-glucose | 1.69 | 0.0453 |
| DHAP/glyceraldehyde 3P | 1.64 | 0.0272 |
| Beta-alanine | 1.59 | 0.039 |
| Lactate | 1.57 | 0.0158 |
| Taurocholate | 1.55 | 0.0176 |
| Glutamate | 1.53 | 0.0141 |
| Malondialdehyde | 1.43 | 0.0057 |
| 2-aminoadipate | 1.39 | 0.0499 |
| Isoleucine | 1.38 | 0.0245 |
| Sorbitol | 1.35 | 0.0371 |
| Leucine | 1.30 | 0.0476 |
| 2-hydroxyglutarate | 1.28 | 0.039 |
| Hexoses (HILIC neg) | 1.26 | 0.0318 |
| Sucrose | 0.87 | 0.0149 |
| Oleylcarnitine | 0.78 | 0.0053 |
| Stearoylcarnitine | 0.71 | 0.0258 |
| Acetylglycine | 0.64 | 0.0335 |
| Carnitine | 0.61 | 0.0197 |
| AMP | 0.61 | 0.0083 |
| Myristoylcarnitine | 0.60 | 0.0001 |
| palmitoylcarnitine | 0.58 | 0.0007 |
| GMP | 0.50 | 0.0208 |
| Butyrobetaine | 0.49 | 0.0002 |
| Propionylcarnitine | 0.47 | 0.0099 |
| Acetylcarnitine | 0.44 | 0.0023 |
| Alpha-glycerophosphate | 0.39 | 0.0302 |
| Malonylcarnitine | 0.37 | 0.0005 |
| 2-deoxycytidine | 0.35 | 0.0232 |
| Lauroylcarnitine | 0.25 | <.0001 |
| Lactose | 0.24 | 0.0197 |
| Butyrylcarnitine/isobutyrylcarnitine | 0.23 | 0.0001 |
| Hexanoylcarnitine | 0.20 | <.0001 |

**Table S11.** Transcriptomic and metabolomic data for 48 BC lines were extracted from public databases.^[41]^ SUV-H-like and SUV-L-like status was assigned to the cell lines by unsupervised hierarchical clustering using PETsign (the list of cell lines and their SUV-like status is shown in Figure S3). Average production of the metabolites was calculated separately for SUV-H-like and SUV-L-like cell lines and the fold-change (FC) SUV-H/SUV-L determined as shown. Red and blue indicate metabolites produced in higher or lower amounts, respectively in SUV-H-like *vs.* SUV-L-like cell lines. Only metabolites displaying significant differences are shown. P-values were determined using the non-parametric Wilcoxon test within JMP.

**Table S12. Prognostic stratification by PETsign and StemPrintER in the METABRIC and IEO clinical cohorts.**

| **Dataset and Analysis** | **Signatures** | **Level 1** | **Level 2** | **HR** | **P** | **95% CI** |
| --- | --- | --- | --- | --- | --- | --- |
| METABRIC  Univariate | PETsign/SP | SUV-H/SP-H | SUV-L/SP-L | 2.82 | <0.0001 | 2.29-3.48 |
|  |  | SUV-H/SP-L | SUV-L/SP-L | 1.73 | 0.0007 | 1.26-2.37 |
|  |  | SUV-H/SP-H | SUV-L/SP-H | 1.68 | <0.0001 | 1.36-2.09 |
|  |  | SUV-L/SP-H | SUV-L/SP-L | 1.68 | <0.0001 | 1.30-2.17 |
|  |  | SUV-H/SP-H | SUV-H/SP-L | 1.64 | 0.0007 | 1.23-2.17 |
|  |  | SUV-H/SP-L | SUV-L/SP-H | 1.03 | 0.87 | 0.75-1.42 |
| METABRIC  Bivariate | SP risk (ALL) | SP-H | SP-L | 1.66 | <0.0001 | 1.37-2.01 |
|  | PETsign risk (ALL) | SUV-H | SUV-L | 1.70 | <0.0001 | 1.42-2.03 |
|  | SP risk (Luminal) | SP-H | SP-L | 1.67 | <0.0001 | 1.35-2.06 |
|  | PETsign risk (Luminal) | SUV-H | SUV-L | 1.64 | <0.0001 | 1.33-2.03 |
| IEO  Univariate | PETsign/SP | SUV-H/SP-H | SUV-L/SP-L | 6.00 | <0.0001 | 3.46-10.44 |
|  |  | SUV-H/SP-L | SUV-L/SP-L | 3.33 | 0.026 | 1.29-8.58 |
|  |  | SUV-L/SP-H | SUV-L/SP-L | 3.13 | 0.0001 | 1.75-5.59 |
|  |  | SUV-H/SP-H | SUV-L/SP-H | 1.91 | 0.0003 | 1.34-2.72 |
|  |  | SUV-H/SP-H | SUV-H/SP-L | 1.80 | 0.16 | 0.78-4.12 |
|  |  | SUV-H/SP-L | SUV-L/SP-H | 1.06 | 0.88 | 0.46-2.48 |
| IEO  Bivariate | SP risk (ALL) | SP-H | SP-L | 2.68 | <0.0001 | 1.65-4.35 |
|  | PETsign risk (ALL) | SUV-H | SUV-L | 2.04 | <0.0001 | 1.45-2.86 |
|  | SP risk (Luminal) | SP-H | SP-L | 2.55 | 0.0003 | 1.54-4.23 |
|  | PETsign risk (Luminal) | SUV-H | SUV-L | 2.56 | <0.0001 | 1.73-3.77 |

**Table S12.** The combined performance of PETsign and StemPrintER (SP) in the METABRIC and 970-IEO cohorts is shown in univariate and bivariate analyses. Hazard ratios (HR) and p-values (P) were calculated with the Cox proportional hazards model using JMP. The 95% confidence intervals (CI) are shown. Significant values are indicated in red.

**SUPPORTING INFORMATION: FIGURES**

**Figure S1. Examples of PET/CT images.** Images are shown of PET/CT images of a low a) and a high b) uptake patient. For each example, the MIP (maximum intensity projection) image with the ROI of the uptake superimposed (left), the axial PET (middle) and the axial PET/CT merged images (right) are shown.

**Figure S2. Additional data to Figure 4 of the main text.** a**)** The pattern of amplification/co-amplification of the 18 genes uncovered in the mining of the TCGA dataset (Figure 4a) was investigated in the METABRIC dataset. In total, 725 BCs (38%) of the METABRIC database harbored co-amplification of the 18 genes (indicated by dark red bars). PETsign genes are in bold, while the other genes belong to the 135-gene signature. Note that no amplification data were retrievable for CXCL8. b) Amplification and overexpression of the 18 genes in the TCGA dataset. The graph shows the result of the combined analysis of amplification and mRNA overexpression in the TCGA dataset: 504 BC cases (~56%) showed one or both alterations. Amplification, dark red, overexpression, light red. For overexpression, we selected the function “mRNA expression z-scores relative to diploid samples (RNA Seq V2 RSEM)” with a threshold z-score of 2.0.

**Figure S3. Additional data to Figures 5 and 6 of the main text.** PETsign was used for hierarchical clustering of 53 BC cell lines with transcriptomic (RNAseq) data available from the Cancer Cell Line Encyclopedia (CCLE) collection (https://sites.broadinstitute.org/ccle/datasets).^[41a]^ Rows represent cell lines, while the columns correspond to genes. For 48 of these cell lines, metabolomics data were also available,^[41b]^ and were used for the clustering shown in Figure 5d.

**Figure S4. Additional data to Figure 6 of the main text.** a) The 13 BC lines are shown, with their molecular subtype, as per reference,^[42]^ and their SUV-like status, as per panel b. Note that the MDA-MB-453 cell line harbors HER2 amplification, yet it shows moderate levels of ERBB2 over-expression (see panel c). b) Principal component analysis (PCA) of the 13 BC cell lines stratified by PETsign. Red, SUV-H-like lines; blue, SUV-L-like lines. c) The immunoblot in Figure 6e is shown again here with additional antibody staining for different EGFR phosphosites (pY992 and pY1068 in addition to pY1086). EGFR, total EGFR; GAPDH and actin, loading controls. Anti-ErbB2 was also performed to verify the HER2 status of the cell lines. MW markers (kDa) are shown. The Ponceau staining of the membrane is shown beneath the blots.

Methods: All cell lines used in this study have been obtained from ATCC and derive from female human subject. MCF-7 (HTB-22), T47D (HTB-133), BT-474 (HTB-20), BT-549 (HTB-122), MDA-MB-231 (HTB-26), and MDA-MB-453 (HTB-131) were cultured in DMEM + 10% FBS + 2 mM L-glutamine. ZR751 (CRL-1500), HCC-1954 (CRL-2338) and HCC-70 (CRL-2315) were cultured in RPMI-1640 + 10% FBS + 2 mM L-glutamine + 1 mM sodium pyruvate + 10 mM HEPES. MDA-MB-468 (HTB-132) were cultured in DMEM/Ham’s F12 (1:1) + 10% FBS + 2 mM L-glutamine. BT-20 (HTB-19) were cultured in MEM with Earle’s Salts + 10% FBS + 2 mM L-glutamine + 1 mM sodium pyruvate + 0.1 mM NEAA. BT-483 (HTB-121) were cultured in RPMI-1640 + 20% FBS + 2 mM L-glutamine + 0.01 mg mL^-1^ insulin. Hs-578T (HTB-126) were cultured in DMEM + 10% FBS + 2 mM L-glutamine + 0.01 mg mL^-1^ insulin.

DMEM, RPMI-1640, MEM, and Ham’s F12 were from: Euroclone #ECM0103L, Thermo Fisher # 21875034, Lonza #LOBE12125F, and Thermo Fisher #31765027, respectively. FBS (fetal bovine serum) was of North American origin (Hyclone, CHA30088L) for MCF-7, T47D, BT-474, MDA-MB-431, HCC-1954, BT-483, HS-578T, HCC-70, MDA-MB-453, and BT-20 or of South America origin (Microtech # RM10432) for ZR-751, BT-549, MDA-MB-468. Supplements were: L-glutamine (Lonza #LOBE17605E), sodium pyruvate (Lonza #LOBE13115E), HEPES (Sigma Aldrich # H0887), Non-Essential Amino Acids (NEAA) (Lonza #LOBE13114E), insulin (Merck Life Science # 91077C). CXCL8 was from R&D (R&D cat. 208-IL-050), EGF was from R&D (cat. 236-EG).

All cell lines, at each batch freezing, were authenticated by STR profiling (StemElite ID System, Promega) and tested for mycoplasma by PCR,^[43]^ and biochemical assay (Aurogene, REP-MYS-100).

**REFERENCES**

[1] W. Wen, D. Xuan, Y. Hu, X. Li, L. Liu, D. Xu, *PLoS One* **2019**, *14* (12), e0225959, https://doi.org/10.1371/journal.pone.0225959.

[2] a) D. Groheux, A. Martineau, L. Teixeira, M. Espie, P. de Cremoux, P. Bertheau, P. Merlet, C. Lemarignier, *Breast Cancer Res* **2017**, *19* (1), 3, https://doi.org/10.1186/s13058-016-0793-2; b) D. Groheux, A. Sanna, M. Majdoub, P. de Cremoux, S. Giacchetti, L. Teixeira, M. Espie, P. Merlet, A. de Roquancourt, D. Visvikis, M. Hatt, M. Resche-Rigon, E. Hindie, *J Nucl Med* **2015**, *56* (6), 824, https://doi.org/10.2967/jnumed.115.154138.

[3] a) H. Yang, Y. H. Geng, P. Wang, H. Q. Zhang, W. G. Fang, X. X. Tian, *Cell Death Dis* **2022**, *13* (3), 199, https://doi.org/10.1038/s41419-022-04647-6; b) M. K. Oehler, D. C. Fischer, M. Orlowska-Volk, F. Herrle, D. G. Kieback, M. C. Rees, R. Bicknell, *Br J Cancer* **2003**, *89* (10), 1927, https://doi.org/10.1038/sj.bjc.6601397.

[4] L. Zhu, N. Ma, B. Wang, L. Wang, C. Zhou, Y. Yan, J. He, Y. Ren, *Cancer Manag Res* **2019**, *11*, 1503, https://doi.org/10.2147/CMAR.S193396.

[5] L. Ye, F. Li, Y. Song, D. Yu, Z. Xiong, Y. Li, T. Shi, Z. Yuan, C. Lin, X. Wu, L. Ren, X. Li, L. Song, *Int J Cancer* **2018**, *143* (10), 2602, https://doi.org/10.1002/ijc.31766.

[6] S. Sridhar, C. Rajesh, P. V. Jishnu, P. Jayaram, S. P. Kabekkodu, *Breast Cancer Res Treat* **2020**, *179* (2), 301, https://doi.org/10.1007/s10549-019-05477-5.

[7] L. Wang, H. Wang, C. Yang, Y. Wu, G. Lei, Y. Yu, Y. Gao, J. Du, X. Tong, F. Zhou, Y. Li, Y. Wang, *Front Genet* **2022**, *13*, 900111, https://doi.org/10.3389/fgene.2022.900111.

[8] a) F. Chen, B. Han, Y. Meng, Y. Han, B. Liu, B. Zhang, Y. Chang, P. Cao, Y. Fan, K. Tan, *Aging (Albany NY)* **2021**, *13* (16), 20438, https://doi.org/10.18632/aging.203427; b) N. Chan, A. Willis, N. Kornhauser, M. M. Ward, S. B. Lee, E. Nackos, B. R. Seo, E. Chuang, T. Cigler, A. Moore, D. Donovan, M. Vallee Cobham, V. Fitzpatrick, S. Schneider, A. Wiener, J. Guillaume-Abraham, E. Aljom, R. Zelkowitz, J. D. Warren, M. E. Lane, C. Fischbach, V. Mittal, L. Vahdat, *Clin Cancer Res* **2017**, *23* (3), 666, https://doi.org/10.1158/1078-0432.CCR-16-1326.

[9] J. Zhang, L. Wang, X. Xu, X. Li, W. Guan, T. Meng, G. Xu, *Front Oncol* **2020**, *10*, 1787, https://doi.org/10.3389/fonc.2020.01787.

[10] a) R. A. de Araujo, F. A. C. da Luz, E. da Costa Marinho, C. P. Nascimento, T. R. Mendes, E. R. T. Mosca, L. de Andrade Marques, P. F. R. Delfino, R. M. Antonioli, A. da Silva, M. L. G. Dos Reis Monteiro, M. B. Neto, M. J. B. Silva, *J Cancer Res Clin Oncol* **2023**, https://doi.org/10.1007/s00432-023-05094-2; b) E. Tsutsumi, J. Stricklin, E. A. Peterson, J. A. Schroeder, S. Kim, *Mol Cell Biol* **2022**, *42* (2), e0038221, https://doi.org/10.1128/MCB.00382-21.

[11] L. Li, Y. P. Gan, H. Peng, *Exp Cell Res* **2022**, *416* (2), 113139, https://doi.org/10.1016/j.yexcr.2022.113139.

[12] a) J. Motyka, E. Gacuta, A. Kicman, M. Kulesza, P. Lawicki, S. Lawicki, *J Clin Med* **2022**, *11* (22), https://doi.org/10.3390/jcm11226694; b) H. Hozhabri, M. M. Moghaddam, M. M. Moghaddam, A. Mohammadian, *Sci Rep* **2022**, *12* (1), 10374, https://doi.org/10.1038/s41598-022-14610-2; c) R. X. Wang, P. Ji, Y. Gong, Z. M. Shao, S. Chen, *Breast Cancer Res Treat* **2020**, *181* (3), 561, https://doi.org/10.1007/s10549-020-05660-z; d) L. Tiainen, M. Hamalainen, T. Luukkaala, M. Tanner, O. Lahdenpera, P. Vihinen, A. Jukkola, P. Karihtala, E. Moilanen, P. L. Kellokumpu-Lehtinen, *Clin Breast Cancer* **2019**, *19* (4), e522, https://doi.org/10.1016/j.clbc.2019.03.006; e) Q. I. Fang, X. Wang, G. Luo, M. Yu, X. Zhang, N. Xu, *Anticancer Res* **2017**, *37* (9), 4845, https://doi.org/10.21873/anticanres.11892.

[13] a) J. R. Sainsbury, J. R. Farndon, G. K. Needham, A. J. Malcolm, A. L. Harris, *Lancet* **1987**, *1* (8547), 1398, https://doi.org/10.1016/s0140-6736(87)90593-9; b) B. D. Lehmann, J. A. Bauer, X. Chen, M. E. Sanders, A. B. Chakravarthy, Y. Shyr, J. A. Pietenpol, *J Clin Invest* **2011**, *121* (7), 2750, https://doi.org/10.1172/JCI45014; c) H. Masuda, D. Zhang, C. Bartholomeusz, H. Doihara, G. N. Hortobagyi, N. T. Ueno, *Breast Cancer Res Treat* **2012**, *136* (2), 331, https://doi.org/10.1007/s10549-012-2289-9; d) R. Bhargava, W. L. Gerald, A. R. Li, Q. Pan, P. Lal, M. Ladanyi, B. Chen, *Mod Pathol* **2005**, *18* (8), 1027, https://doi.org/10.1038/modpathol.3800438.

[14] K. H. Song, M. S. Park, T. S. Nandu, S. Gadad, S. C. Kim, M. Y. Kim, *Nat Commun* **2016**, *7*, 13796, https://doi.org/10.1038/ncomms13796.

[15] A. Nagelkerke, H. Mujcic, J. Bussink, B. G. Wouters, H. W. van Laarhoven, F. C. Sweep, P. N. Span, *Cancer* **2011**, *117* (16), 3670, https://doi.org/10.1002/cncr.25938.

[16] E. Jacquet, F. Chuffart, A. L. Vitte, E. Nika, M. Mousseau, S. Khochbin, S. Rousseaux, E. Bourova-Flin, *BMC Genomics* **2023**, *24* (1), 463, https://doi.org/10.1186/s12864-023-09571-3.

[17] a) C. Liu, J. Cao, S. Lin, Y. Zhao, M. Zhu, Z. Tao, X. Hu, *Onco Targets Ther* **2020**, *13*, 8735, https://doi.org/10.2147/OTT.S256970; b) R. Liao, G. Ren, H. Liu, X. Chen, Q. Cao, X. Wu, J. Li, C. Dong, *Sci Rep* **2018**, *8* (1), 16743, https://doi.org/10.1038/s41598-018-35106-y.

[18] Y. Yuan, H. Gao, Y. Zhuang, L. Wei, J. Yu, Z. Zhang, L. Zhang, L. Wang, *Ther Adv Med Oncol* **2021**, *13*, 17588359211027836, https://doi.org/10.1177/17588359211027836.

[19] M. Suleman, A. Chen, H. Ma, S. Wen, W. Zhao, D. Lin, G. Wu, Q. Li, *Cell Cycle* **2019**, *18* (21), 2914, https://doi.org/10.1080/15384101.2019.1662259.

[20] a) B. H. Choi, V. Rawat, J. Hogstrom, P. A. Burns, K. O. Conger, M. E. Ozgurses, J. M. Patel, T. S. Mehta, A. Warren, L. M. Selfors, T. Muranen, J. L. Coloff, *Cell Rep* **2022**, *38* (3), 110278, https://doi.org/10.1016/j.celrep.2021.110278; b) G. D. Barnabas, J. S. Lee, T. Shami, M. Harel, L. Beck, M. Selitrennik, L. Jerby-Arnon, N. Erez, E. Ruppin, T. Geiger, *Cancer Res* **2021**, *81* (6), 1443, https://doi.org/10.1158/0008-5472.CAN-19-3020; c) T. De Marchi, M. A. Timmermans, A. M. Sieuwerts, M. Smid, M. P. Look, N. Grebenchtchikov, F. Sweep, J. G. Smits, V. Magdolen, C. H. M. van Deurzen, J. A. Foekens, A. Umar, J. W. Martens, *Sci Rep* **2017**, *7* (1), 2099, https://doi.org/10.1038/s41598-017-02296-w.

[21] X. Wang, H. Saso, T. Iwamoto, W. Xia, Y. Gong, L. Pusztai, W. A. Woodward, J. M. Reuben, S. L. Warner, D. J. Bearss, G. N. Hortobagyi, M. C. Hung, N. T. Ueno, *Cancer Res* **2013**, *73* (21), 6516, https://doi.org/10.1158/0008-5472.CAN-13-0967.

[22] a) Y. L. Zhang, L. Deng, L. Liao, S. Y. Yang, S. Y. Hu, Y. Ning, F. L. Zhang, D. Q. Li, *Cell Death Dis* **2022**, *13* (4), 408, https://doi.org/10.1038/s41419-022-04785-x; b) Z. Li, O. McGinn, Y. Wu, A. Bahreini, N. M. Priedigkeit, K. Ding, S. Onkar, C. Lampenfeld, C. A. Sartorius, L. Miller, M. Rosenzweig, O. Cohen, N. Wagle, J. K. Richer, W. J. Muller, L. Buluwela, S. Ali, T. C. Bruno, D. A. A. Vignali, Y. Fang, L. Zhu, G. C. Tseng, J. Gertz, J. M. Atkinson, A. V. Lee, S. Oesterreich, *Nat Commun* **2022**, *13* (1), 2011, https://doi.org/10.1038/s41467-022-29498-9; c) J. Li, X. Shu, J. Xu, S. M. Su, U. I. Chan, L. Mo, J. Liu, X. Zhang, R. Adhav, Q. Chen, Y. Wang, T. An, X. Zhang, X. Lyu, X. Li, J. H. Lei, K. Miao, H. Sun, F. Xing, A. Zhang, C. Deng, X. Xu, *Nat Commun* **2022**, *13* (1), 1481, https://doi.org/10.1038/s41467-022-29151-5; d) J. Y. Goh, M. Feng, W. Wang, G. Oguz, S. Yatim, P. L. Lee, Y. Bao, T. H. Lim, P. Wang, W. L. Tam, A. R. Kodahl, M. B. Lyng, S. Sarma, S. Y. Lin, A. Lezhava, Y. S. Yap, A. S. T. Lim, D. S. B. Hoon, H. J. Ditzel, S. C. Lee, E. Y. Tan, Q. Yu, *Nat Med* **2017**, *23* (11), 1319, https://doi.org/10.1038/nm.4405.

[23] J. Ma, Z. He, H. Zhang, W. Zhang, S. Gao, X. Ni, *Cell Death Dis* **2021**, *12* (6), 550, https://doi.org/10.1038/s41419-021-03797-3.

[24] a) E. Oliemuller, R. Newman, S. M. Tsang, S. Foo, G. Muirhead, F. Noor, S. Haider, I. Aurrekoetxea-Rodriguez, M. D. Vivanco, B. A. Howard, *Elife* **2020**, *9*, https://doi.org/10.7554/eLife.58374; b) E. Oliemuller, N. Kogata, P. Bland, D. Kriplani, F. Daley, S. Haider, V. Shah, E. J. Sawyer, B. A. Howard, *J Pathol* **2017**, *243* (2), 193, https://doi.org/10.1002/path.4939.

[25] a) A. Gothlin Eremo, K. Lagergren, L. Othman, S. Montgomery, G. Andersson, E. Tina, *Sci Rep* **2020**, *10* (1), 1451, https://doi.org/10.1038/s41598-020-58323-w; b) K. Walaszek, E. E. Lower, P. Ziolkowski, G. F. Weber, *Br J Cancer* **2018**, *119* (10), 1259, https://doi.org/10.1038/s41416-018-0228-1; c) J. Insua-Rodriguez, M. Pein, T. Hongu, J. Meier, A. Descot, C. M. Lowy, E. De Braekeleer, H. P. Sinn, S. Spaich, M. Sutterlin, A. Schneeweiss, T. Oskarsson, *EMBO Mol Med* **2018**, *10* (10), https://doi.org/10.15252/emmm.201809003; d) S. Sangaletti, C. Tripodo, S. Sandri, I. Torselli, C. Vitali, C. Ratti, L. Botti, A. Burocchi, R. Porcasi, A. Tomirotti, M. P. Colombo, C. Chiodoni, *Cancer Res* **2014**, *74* (17), 4706, https://doi.org/10.1158/0008-5472.CAN-13-3334.

[26] P. Y. Chu, Y. T. Tzeng, K. H. Tsui, C. Y. Chu, C. J. Li, *Aging (Albany NY)* **2022**, *14* (5), 2252, https://doi.org/10.18632/aging.203933.

[27] D. Jeong, J. Ham, H. W. Kim, H. Kim, H. W. Ji, S. H. Yun, J. E. Park, K. S. Lee, H. Jo, J. H. Han, S. Y. Jung, S. Lee, E. S. Lee, H. S. Kang, S. J. Kim, *Am J Cancer Res* **2021**, *11* (6), 2568.

[28] R. Bradbury, W. G. Jiang, Y. X. Cui, *Anticancer Res* **2016**, *36* (3), 1143.

[29] H. N. Li, X. R. Li, Z. T. Lv, M. M. Cai, G. Wang, Z. F. Yang, *Cancer Med* **2020**, *9* (24), 9554, https://doi.org/10.1002/cam4.3543.

[30] H. Cohen-Dvashi, N. Ben-Chetrit, R. Russell, S. Carvalho, M. Lauriola, S. Nisani, M. Mancini, N. Nataraj, M. Kedmi, L. Roth, W. Kostler, A. Zeisel, A. Yitzhaky, J. Zylberg, G. Tarcic, R. Eilam, Y. Wigelman, R. Will, S. Lavi, Z. Porat, S. Wiemann, S. Ricardo, F. Schmitt, C. Caldas, Y. Yarden, *EMBO Mol Med* **2015**, *7* (3), 299, https://doi.org/10.15252/emmm.201404134.

[31] W. Lou, J. Liu, B. Ding, L. Jin, L. Xu, X. Li, J. Chen, W. Fan, *Aging (Albany NY)* **2019**, *11* (9), 2628, https://doi.org/10.18632/aging.101934.

[32] a) S. Di, R. Bai, D. Lu, C. Chen, T. Ma, Z. Zou, Z. Zhang, *Cell Death Discov* **2022**, *8* (1), 249, https://doi.org/10.1038/s41420-022-01043-z; b) X. Niu, Y. Zhan, S. Zhang, Z. Liu, C. Qu, *Biophys Rep* **2021**, *7* (3), 185, https://doi.org/10.52601/bpr.2021.210002; c) T. Z. Parris, A. Danielsson, S. Nemes, A. Kovacs, U. Delle, G. Fallenius, E. Mollerstrom, P. Karlsson, K. Helou, *Clin Cancer Res* **2010**, *16* (15), 3860, https://doi.org/10.1158/1078-0432.CCR-10-0889.

[33] C. Pich-Bavastro, L. Yerly, J. Di Domizio, S. Tissot-Renaud, M. Gilliet, F. Kuonen, *Clin Cancer Res* **2023**, https://doi.org/10.1158/1078-0432.CCR-23-0219.

[34] a) B. Li, E. Severson, J. C. Pignon, H. Zhao, T. Li, J. Novak, P. Jiang, H. Shen, J. C. Aster, S. Rodig, S. Signoretti, J. S. Liu, X. S. Liu, *Genome Biol* **2016**, *17* (1), 174, https://doi.org/10.1186/s13059-016-1028-7; b) T. Li, J. Fan, B. Wang, N. Traugh, Q. Chen, J. S. Liu, B. Li, X. S. Liu, *Cancer Res* **2017**, *77* (21), e108, https://doi.org/10.1158/0008-5472.CAN-17-0307.

[35] J. Racle, K. de Jonge, P. Baumgaertner, D. E. Speiser, D. Gfeller, *Elife* **2017**, *6*, https://doi.org/10.7554/eLife.26476.

[36] F. Finotello, C. Mayer, C. Plattner, G. Laschober, D. Rieder, H. Hackl, A. Krogsdam, Z. Loncova, W. Posch, D. Wilflingseder, S. Sopper, M. Ijsselsteijn, T. P. Brouwer, D. Johnson, Y. Xu, Y. Wang, M. E. Sanders, M. V. Estrada, P. Ericsson-Gonzalez, P. Charoentong, J. Balko, N. de Miranda, Z. Trajanoski, *Genome Med* **2019**, *11* (1), 34, https://doi.org/10.1186/s13073-019-0638-6.

[37] B. Chen, M. S. Khodadoust, C. L. Liu, A. M. Newman, A. A. Alizadeh, *Methods Mol Biol* **2018**, *1711*, 243, https://doi.org/10.1007/978-1-4939-7493-1_12.

[38] C. Curtis, S. P. Shah, S. F. Chin, G. Turashvili, O. M. Rueda, M. J. Dunning, D. Speed, A. G. Lynch, S. Samarajiwa, Y. Yuan, S. Graf, G. Ha, G. Haffari, A. Bashashati, R. Russell, S. McKinney, M. Group, A. Langerod, A. Green, E. Provenzano, G. Wishart, S. Pinder, P. Watson, F. Markowetz, L. Murphy, I. Ellis, A. Purushotham, A. L. Borresen-Dale, J. D. Brenton, S. Tavare, C. Caldas, S. Aparicio, *Nature* **2012**, *486* (7403), 346, https://doi.org/10.1038/nature10983.

[39] N. Cancer Genome Atlas, *Nature* **2012**, *490* (7418), 61, https://doi.org/10.1038/nature11412.

[40] a) S. Pece, D. Disalvatore, D. Tosoni, M. Vecchi, S. Confalonieri, G. Bertalot, G. Viale, M. Colleoni, P. Veronesi, V. Galimberti, P. P. Di Fiore, *EBioMedicine* **2019**, *42*, 352, https://doi.org/10.1016/j.ebiom.2019.02.036; b) S. Pece, I. Sestak, F. Montani, M. Tillhon, P. Maisonneuve, S. Freddi, K. Chu, M. Colleoni, P. Veronesi, D. Disalvatore, G. Viale, R. Buus, J. Cuzick, M. Dowsett, P. P. Di Fiore, *Eur J Cancer* **2022**, *164*, 52, https://doi.org/10.1016/j.ejca.2022.01.003.

[41] a) M. Ghandi, F. W. Huang, J. Jane-Valbuena, G. V. Kryukov, C. C. Lo, E. R. McDonald, 3rd, J. Barretina, E. T. Gelfand, C. M. Bielski, H. Li, K. Hu, A. Y. Andreev-Drakhlin, J. Kim, J. M. Hess, B. J. Haas, F. Aguet, B. A. Weir, M. V. Rothberg, B. R. Paolella, M. S. Lawrence, R. Akbani, Y. Lu, H. L. Tiv, P. C. Gokhale, A. de Weck, A. A. Mansour, C. Oh, J. Shih, K. Hadi, Y. Rosen, J. Bistline, K. Venkatesan, A. Reddy, D. Sonkin, M. Liu, J. Lehar, J. M. Korn, D. A. Porter, M. D. Jones, J. Golji, G. Caponigro, J. E. Taylor, C. M. Dunning, A. L. Creech, A. C. Warren, J. M. McFarland, M. Zamanighomi, A. Kauffmann, N. Stransky, M. Imielinski, Y. E. Maruvka, A. D. Cherniack, A. Tsherniak, F. Vazquez, J. D. Jaffe, A. A. Lane, D. M. Weinstock, C. M. Johannessen, M. P. Morrissey, F. Stegmeier, R. Schlegel, W. C. Hahn, G. Getz, G. B. Mills, J. S. Boehm, T. R. Golub, L. A. Garraway, W. R. Sellers, *Nature* **2019**, *569* (7757), 503, https://doi.org/10.1038/s41586-019-1186-3; b) H. Li, S. Ning, M. Ghandi, G. V. Kryukov, S. Gopal, A. Deik, A. Souza, K. Pierce, P. Keskula, D. Hernandez, J. Ann, D. Shkoza, V. Apfel, Y. Zou, F. Vazquez, J. Barretina, R. A. Pagliarini, G. G. Galli, D. E. Root, W. C. Hahn, A. Tsherniak, M. Giannakis, S. L. Schreiber, C. B. Clish, L. A. Garraway, W. R. Sellers, *Nat Med* **2019**, *25* (5), 850, https://doi.org/10.1038/s41591-019-0404-8.

[42] G. Jiang, S. Zhang, A. Yazdanparast, M. Li, A. V. Pawar, Y. Liu, S. M. Inavolu, L. Cheng, *BMC Genomics* **2016**, *17 Suppl 7* (Suppl 7), 525, https://doi.org/10.1186/s12864-016-2911-z.

[43] C. C. Uphoff, H. G. Drexler, *In Vitro Cell Dev Biol Anim* **2002**, *38* (2), 79, https://doi.org/10.1290/1071-2690(2002)038<0079:CPAFDO>2.0.CO;2.
